# Mitochondrial maturation drives germline stem cell differentiation in *Caenorhabditiselegans*

**DOI:** 10.1038/s41418-019-0375-9

**Published:** 2019-06-19

**Authors:** Nikolaos Charmpilas, Nektarios Tavernarakis

**Affiliations:** 1 Institute of Molecular Biology and Biotechnology, Foundation for Research and Technology-Hellas, Heraklion, Greece; 2 Department of Biology, University of Crete, Heraklion, Greece; 3 Department of Basic Sciences, School of Medicine, University of Crete, 70013 Heraklion, Crete Greece

**Keywords:** Cell biology, Ageing

## Abstract

The *C. elegans* germline recapitulates mammalian stem cell niches and provides an effective platform for investigating key aspects of stem cell biology. However, the molecular and physiological requirements for germline stem cell homeostasis remain largely elusive. Here, we report that mitochondrial biogenesis and function are crucial for germline stem cell identity. We show that general transcription activity in germline mitochondria is highly compartmentalized, and determines mitochondrial maturation. RPOM-1, the mitochondrial RNA polymerase, is differentially expressed as germ nuclei progress from the distal to the proximal gonad arm to form oocytes. Mitochondria undergo changes from globular to tubular morphology and become polarized, as they approach the proximal gonad arm. Notably, this mitochondrial maturation trajectory is evolutionarily conserved. We find that a similar transition and temporal mitochondrial RNA polymerase expression profile characterizes differentiation of mammalian stem cells. In *C. elegans*, ATP, and ROS production increases sharply during maturation. Impaired mitochondrial bioenergetics causes gonad syncytium tumor formation by disrupting the balance between mitosis and differentiation to oocytes, which results in a marked reduction of fecundity. Consequently, compensatory apoptosis is induced in the germline. Sperm-derived signals promote mitochondrial maturation and proper germ cell differentiation via the MEK/ERK kinase pathway. Germ cell fate decisions are determined by a crosstalk between Insulin/IGF-1 and TGF-β signaling, mitochondria and protein synthesis. Our findings demonstrate that mitochondrial transcription activity determines a shift in mitochondrial bioenergetics, which in turn regulates germline stem cell survival and differentiation. Perturbation of mitochondrial transcription hinders proper germ cell differentiation and causes germline tumor development.

## Introduction

In adult *Caenorhabditis elegans* animals, somatic cells are postmitotic and terminally differentiated. Yet, adult hermaphrodite nematodes possess a germ cell population undergoing rapid proliferation. Germ cells are topologically isolated from surrounding somatic tissues and are enclosed in two *U*-shaped gonads. At the distal tip each gonad, the distal tip cell (DTC), a somatic cell of mesenchymal origin, preserves the mitotic identity of nearby germ cell nuclei through GLP-1 (Notch)/LAG-2 (Delta) signaling. The DTC forms a plexus that surrounds adjacent nuclei [1]. The majority of germline nuclei divide once within the proliferative region [2]. Upon escaping from DTC’s vicinity, they invariably progress toward meiosis I (pachytene, diplotene, and diakinesis), become enclosed by a cell membrane after the gonad turn and ultimately form oocytes, which are fertilized upon reaching the spermatheca in the proximal arm [3, 4]. The *C. elegans* germline shares several features analogous to mammalian stem cell niches; thus, providing an effective platform toward the delineation of cellular and molecular mechanisms underlying stem cell fate [5].

A distinguishing characteristic of stem cells relates to energy metabolism and mitochondrial function (reviewed in [6]). Mitochondria originate from endosymbiotic events of ancestral eukaryotic cells with once free-living proteobacteria [7]. Albeit they principally rely on nuclear gene products for their function, they are considered semiautonomous, since they retain their own genome (mtDNA). mtDNA mutations have been associated with diverse human diseases [8]. The human mtDNA encodes 13 electron transport chain (ETC) components, as well as two rRNAs (12S and 16S rRNAs) and the whole repertoire of tRNA molecules [9]. These untranslated RNA species, together with exclusively nuclear-encoded mitochondrial ribosomal proteins, form the 55S mitochondrial ribosome, which synthesizes mtDNA-derived ETC components in the organelle [10]. Transcription of the mitochondrial genome is mediated by a tripartite complex, comprising a dedicated RNA polymerase and two auxiliary transcription factors, TFAM and TFB2M [11–14].

Although mitochondria have been implicated in the maintenance of stemness and the progression toward differentiation, the contribution of the mitochondrial genome remains elusive. To gain relevant insight, we examined the involvement of mitochondrial genome expression in *C. elegans* germ cell homeostasis. We find that perturbations in general mitochondrial transcription and mitochondrial bioenergetics reduces fecundity and causes sterility. Depletion of RPOM-1, the homolog of the mammalian mitochondrial RNA polymerase, generates a tumor phenotype in the pachytene gonad region due to impaired germ cell differentiation, and leads to gonad collapse at elevated temperatures. Induction of apoptosis acts in a protective, compensatory manner to restrict tumor size. Gonadal mitochondrial biogenesis is coupled with extrinsic Insulin/IGF-1 and TGF-β signaling pathways that converge on the germline. Attenuation of general protein synthesis in the germline alleviates the sequelae of RPOM-1 deficiency. Expression of mitochondrial RNA polymerase gradually increases in the course of germ cell differentiation, concurrent with morphological and functional adaptations, associated with enhanced metabolic activity of germline mitochondria. These adaptations are accompanied by increased ATP and ROS production in the proximal gonad arm. Notably, upregulation of mitochondrial RNA polymerase expression, the consequent establishment of tubular mitochondrial morphology and associated bioenergetic profile are evolutionarily conserved during mouse stem cell differentiation. Thus, coordinated expression of the mitochondrial genome is a key component of a regulatory program that determines germ cell differentiation.

## Materials and methods

### Strains and genetics

We followed standard procedures for maintaining *C. elegans* strains. Rearing temperature was set at 20 °C for most of our experiments unless noted otherwise, but was raised at 25 °C when germline expression was assessed to avoid transgene silencing that occurs at lower temperatures. The following nematode strains, which are available from CGC, were utilized in this study: N2: wild-type Bristol isolate, CU5991: *fzo-1(tm1133)* II, CU6372: *drp-1(1108)* IV, and DA631: *eat-3(ad426)* II; and *him-8(e1489)* IV, MT7686: *ced-9(n2812)*/qC1 [*dpy-19(e1259)* *glp-1(q339)*] III, MT1522: *ced-3(n717)* IV, CB1370: *daf-2(e1370)* III, DR40: *daf-1(m40)* IV, RB1206: *rsks-1(ok1255)* III, KX80: *ife-5(ok1934)* II, and CB4037: *glp-1(e2141ts)* III. We used the JJ1850: *unc-119(ed3) III*; *Is*[his-72(1kb 5′UTR)::his-72::SRPVAT::GFP::his-72 (1KB 3'UTR)] and the RW10226: *Is*[p_*pie-1*_mCherry::H2B::*pie-1* 3′UTR]; *Is*[p*his-72*HIS-24::mCherry::let-858 3′UTR] strains for monitoring germline nuclei in vivo, the AD189: *unc-119(ed3) III*; *Is*[p_*pie-1*_GFP::EGG-1+ unc-119(+)] strain for monitoring oocyte membranes in vivo and the OD95: *unc-119(ed3) III*; *Is*[p_*pie-1*_mCherry::HIS-58; p_*pie-1*_GFP::PH(PLC1delta1) +unc-119(+)] strain for simultaneously monitoring oocyte membranes and germline nuclei in vivo. JK4472: WT; *Is*[p_*lag-2*_MYR::tdTomato + p_*ttx-3*_GFP] and JK4475: WT; *Is*[p_*lag-2*_MYR::GFP + p_*ttx-3*_DsRed] were used for monitoring the DTC’s membrane plexus, while DG1575: WT; *Is*[p_*lim-7*_GFP + rol-6(su1006)] was used for monitoring gonad sheath morphology. All the experiments were performed using day 1 (D1) gravid adult animals that were precisely synchronized at the L4 larval stage one day earlier. For *glp-1(e2141)* mutants specifically, we placed eggs on plates at the restrictive temperature of 25 °C for 48 h. Then we transferred L4 larvae on fresh control or *rpom-1(RNAi)* plates and we placed them for one more day at 20 °C prior to nuclear staining and microscopic observation. Culture of *glp-1(e2141)* mutants at 25 °C for 48 h during this developmental window renders *glp-1(e2141)* mutants completely sterile [15]. The following strains were generated in the current study: IR1677: WT; *Ex*[p_*CEOP1740*_RPOM-1::GFP::*unc-54* 3′UTR], IR1968: *unc-119(ed3) III*; *Ex*[p_*CEOP1740*_RPOM-1::GFP::*rpom-1* 3′UTR +unc-119(+)], IR1966: *unc-119(ed3) III*; and *Is*[p_*pie-1*_Perceval::*tbb-2* 3′UTR +unc-119(+)].

### Molecular cloning

Gene inactivation was achieved by bacterial feeding of *C. elegans* with RNAi clones expressing double-stranded RNA targeting the gene of interest. All RNAi treatments were performed continuously from hatching (L1 larval stage) till the day of observation (D1). The following primers were used for generation of RNAi constructs used in the present study. For *rpom-1(RNAi)*: 5′-ATGAGAAGACTGGAACGAATTGTC-3′ and 5′-TAGTGTTCAATCCCTCACCAATC-3′, for *hmg-5(RNAi)*: 5′-GGATCCAATGTTGGGAACAATTTC-3′ and 5′-ACCGGTGGTTGATCTGCATTTTC-3′, for *tfbm-1(RNAi)*: 5′-ATGGCTTCTGCTTCACGTCTCC-3′ and 5′-CATCTTAGGCTCTGCCACGTATTG-3′, for *gld-1(RNAi)*: 5′-CACTCCAACTTACGGTGTTTCG-3′ and 5′-TCTACCGACGAAGTTATACTGAAATG-3′, for *mpk-1(RNAi)*: FW: 5′-GTTCATGGGCAACTTTTTGAG-3′ and 5′-GATTACTGAGCATTTCTGCGAG-3′, for *mek-2(RNAi)*: 5′-CGTAATCCGTTGGGACTCAG-3′ and 5′-CGAGAATCCTGCGAGAACTG-3′, for *goa-1(RNAi)*: 5′-CCACATACAGTGAGTGAGTAGAG-3′ and 5′-CTCTCTGTCAGCCGAACC-3′, and for *gsa-1(RNAi)*: 5′-AGCAAAAAAGAACGAGCAAC-3′ and 5′-TGTCCTCCCAGAGTACAAGA-3′.

For cloning the minimal 2.1 kb operon promoter upstream of CEOP1740 operon, which contains the *rpom-1* gene, we used the 5′-CCATGAAATTGAGGATTCTGAAAC-3′ and 5′- ATTTCCGTTTAGTAGCGATTTTTAACAG-3′ primer pair. The corresponding product was inserted in TOPO and then in pPD95.77 linearized with PstI-BamHI digestion. For cloning the 3.5 kb *rpom-1* full length cDNA, we utilized *C. elegans* total RNA as a template to synthesize the *rpom-1* single-strand cDNA with the PrimeScript™ Reverse Transcriptase kit (Taqara) and the 5′-ACCGGTGGACTAAAAAAATAAACAGAATCCTTGAC-3′ gene-specific reverse primer. Then, the previous primer was used in the same reaction with the 5′-GGATCCATGAGAAGACTGGAACGAATTGTC-3′ forward primer to synthesize the full length *rpom-1* cDNA. Expand high-fidelity DNA polymerase (Roche) was used in the PCR reaction to minimize errors. The amplified fragment was inserted upstream of GFP in the pPD95.77 expression vector linearized with BamHI-AgeI restriction digestion. For amplifying the 0.1 kb *rpom-1* 3′UTR fragment, we used the 5′-GAATTCAGTTAGAAGTGTTTTTTTTGTTG-3′ and 5′-GGGCCCCATTTTCTGATTCCAGCG-3′ pair of primers. Upon ligation into TOPO vector, we isolated the corresponding fragment using EcoRI-ApaI restriction digestion. This was then inserted in pPD95.77 linearized with EcoRI-ApaI digestion downstream of the *rpom-1* cDNA::GFP cassette to replace the *unc-54* 3′UTR fragment. For creating the *pie-1* promoter Perceval::tbb-2 3′UTR plasmid, we extracted the Perceval from the pRsetB-his7-Perceval plasmid (catalog number 20336, Addgene) using XbaI-XhoI digestion. In parallel, we amplified the tbb-2 3′UTR using the 5′-CTCGAGCAATAAATGCAAGATCCTTTCAAGC-3′ and 5′-GAATTCTTTCCTCTTTTTGTTGGGTCACTC-3′ pair of primers. We digested pPD95.77 with XbaI-EcoRI digestion to remove GFP and we simultaneously ligated the two inserts with the linearized vector in a single reaction. An extended *pie-1* promoter was amplified using the 5′-GTCGACTCGTATTTCTCAGTCATTTTTGTG-3′ and 5′-CCATGGATCGTTTTGTATTCTGTGTGCTGG-3′ primer pair and was inserted upstream of the Perceval::tbb-2 cassette using SalI-NcoI restriction sites.

### Nematode strain generation

We used both microinjection into *C. elegans* gonads and bombardment with gold nanoparticles to generate transgenic strains. To avoid undesired transgene silencing in the germline, in microinjections we used low micromolar concentrations (5 ng/μL) of both the reporter plasmid and the cotransformation marker and an excess of PvuII-digested *E. coli* genomic fragments (50 ng/μL), as previously described [16].

### Mitochondrial DNA quantification

mtDNA was quantified by quantitative real-time PCR-based method as previously described [17]. The 5′-GTTTATGCTGCTGTAGCGTG-3′ and 5′-CTGTTAAAGCAAGTGGACGAG-3′ primer pair was used for measuring mtDNA levels. These primers hybridize in the *atp-6* gene which is located in the mitochondrial genome (mtDNA). The results were normalized to genomic DNA amplified with the 5′-TGGAACTCTGGAGTCACACC-3′ and 5′-CATCCTCCTTCATTGAACGG-3′ primer pair, which hybridizes to the genomic region of *ama-1* gene. Quantitative PCR was performed using a Bio-Rad CFX96 real-time PCR system, and was repeated three times.

### Measurements of mitochondrial activity

Staining with mitochondrial dyes (TMRE, Mitotracker ROS) to assess functional mitochondria and ATP measurements in whole animals were performed as previously described [18].

### mRNA quantification

Total RNA from synchronized D4 animals was extracted using the TRIzol reagent (Invitrogen). The following sets of real-time primers were used in the current study: for measuring *hmg-5* mRNA levels: FW: 5′-CGTCCAAGTGTTCCTCCAAGTG-3′ and 5′-CTTCGCTTCGTCTGTGTACTTCTTT-3′, for measuring *tfbm-1* mRNA levels: 5′-CACAAGAAAGATAGCAAAACACGC-3′ and 5′-CGAGATGCTGTAACGGCGG-3′, and for measuring *rpom-1* mRNA levels: 5′-GGTGTCGGCTGGTATCCTCAAC-3′ and 5′-TGGCACAATCTCCTGAGTAGCC-3′.

### Egg laying assays

For assessing egg laying, we placed single, precisely synchronized gravid adult nematodes (at D1–D2 of adulthood) in individual 33 mm NGM plates seeded with a standard OP50 bacterial lawn. The number of eggs that each adult worm produced in the next 12 h were measured. At least 20 individual measurements were acquired per experimental condition. The egg laying assays were performed at 20 °C, apart from the one corresponding to the mitochondrial dynamics mutants (*fzo-1*, *drp-1*, and *eat-3*) that were performed at 25 °C.

### Antimycin A treatment

Antimycin A (catalogue number A8674, Sigma-Aldrich) was supplemented on top of NGM plates seeded with OP50 *E. coli* bacterial food at a final concentration of 50 nM per plate. The plates were previously placed in a UV-crosslinker for 15 min to kill off bacteria before Antimycin A addition. L4-staged nematodes were placed on Antimycin A-containing plates for 2 days prior to microscopic observation. The entire experiment was performed at 25 °C to avoid undesired silencing of the Perceval-expressing transgene.

### Nuclear staining of nematodes

We synchronized adult worms of the genotypes of interest at the first day of adulthood (D1). We stained with Hoechst 33342 to monitor germline nuclei at this stage. First we washed NGM plates with M9 buffer and collected the animals in a 1.5 mL Eppendorf tube. We let the worms pellet with gravity and we washed once with PBS-Tween20^®^ 0.01%. We centrifuged at 3000 rpm for 1 min to pellet the animals and we removed the supernatant. Fixation was performed with cold methanol 100% for maximum 5 min at −20 °C. The Eppendorf tubes were spinned and the fixative was removed. Upon fixation, cell membranes were permeated by washing once with PBS-Tween20^®^ 0.1%. Upon centrifugation at 3000 rpm for 1 min, the worm pellet was stained by adding 300 μL of diluted Hoechst 33342 solution (final concentration 1 μg/mL) for 5 min in the dark. The Eppendorf tubes were centrifuged, the supernatant was removed and the pellet was washed for a final time with PBS-Tween20^®^ 0.1% to remove excessive Hoechst stain. The supernatant was aspirated; 20 μL 80% glycerol was added in each Eppendorf tube and the samples were mounted in microscopic slides prior to observation. The transition zone nuclei were distinguished due to the polarized localization of chromosomes in germ nuclei [19].

### Immunofluorescence of *C. elegans* gonads

A large number of synchronized D1 adult worms grown on NGM plates were washed two repetitive times with M9 buffer and 20 μL of 20 mM levamisole was added on the worm pellet to anaesthetize the animals. About 7–8 μL of the mixture was placed on top of polylysine-treated slides and the animals were dissected using a 8 mm insulin syringe, until the gonads were released from the animals due to mechanical pressure. The extracted tissue was fixed with 4% paraformaldehyde in PBS for 20 min at room temperature. The fixative was removed by washing the specimen twice with PBS. Then, the gonads were permeabilized with 0.2% Triton X-100 in PBS 1× for 5 min and rinsed twice with PBS. Next, the tissue was incubated in blocking solution (1% BSA in PBS-Tween 0.02%) for 1h at room temperature. The incubation with the rabbit anti-phospho-Histone H3 (Serine 10) (06-570, Sigma-Aldrich) antibody (diluted 1:300 in blocking solution) was performed overnight in the dark at 4 °C and the slides were sealed to avoid evaporation. The sample was washed thrice with PBS-Tween 0.02% to remove the primary antibody excess. Finally, the gonads were incubated for 1 h with the anti-rabbit IgG AlexaFluor^®^ 488 (catalogue number ab150077, Abcam) secondary antibody (diluted 1:500 in PBS-Tween 0.02%) and DAPI (final concentration 2 μg/mL) to stain germline nuclei, rinsed three times with PBS and mounted with glycerol prior to observation.

### Staining germline mitochondria in live animals

To stain germline mitochondria, mitochondrial dyes were added on top of standard NGM plates seeded with OP50 *E. coli* bacterial food. The plates were previously placed in a UV-crosslinker for 15 min to kill off bacteria and avoid undesired catabolism of compounds. TMRE (Tetramethylrhodamine, ethyl ester, perchlorate, catalog number T-669; Molecular Probes, Invitrogen) and Mitotracker Red CM-H2X ROS (catalog number M-7513; Molecular Probes, Invitrogen) were administered by food in a final concentration of 1 μΜ per plate and DIOC6(3) (3,3′-Dihexyloxacarbocyanine Iodide, CAS Number 53213-82-4, Sigma-Aldrich) in a final concentration of 2 μΜ per plate. The plates were allowed to dry, constantly protected from light, and then wild-type eggs harvested from hypochlorite-treated, well-fed adult animals were placed on top of the bacterial lawn. The larvae that hatched were fed with the dyes for three consecutive days at 25 °C (when His-72::GFP transgenic animals were used) or for four consecutive days at 20 °C (when wild-type animals were used), until they produced D1–2 gravid adult nematodes. Adult animals were anaesthetized with 20 mM levamisole and were microscopically examined using a ZEISS LSM 710 confocal microscope.

### Mammalian cell culture and immunofluorescence

Standard cell culture procedures were followed. J1 embryonic stem cells were initially cultured for four passages on top of Mitomycin C-treated mouse fibroblasts and then on gelatin in LIF cytokine-containing ES medium to preserve their pluripotent identity. Large globular ES colonies were acquired. LIF was removed and the ES medium was replaced with EB medium to favor unbiased differentiation toward multiple cell lineages. Cells were grown for up to 48 h on EB medium on 12-well plates on top of cover slips. For immunofluorescence experiments, cells were washed three times with PBS before they were fixed with 4% paraformaldehyde in RT. The cover slips were washed again three times with PBS. The blocking was performed with 0.2% Triton X, 10% FBS for 1 h. Mouse monoclonal anti-MTCO1 (catalogue number ab14705, Abcam), rabbit polyclonal anti-POLRMT (catalogue number PA5-28196, Thermo Scientific), and mouse monoclonal anti-OCT4 (catalogue number sc-5279, Santa Cruz Biotechnology) were used as primary antibodies according to the manufacturer’s instructions. The appropriate primary antibody combination was added in blocking solution and was incubated overnight with the specimen at 4 °C. Next day, the specimen was rinsed thrice with PBS and was incubated with anti-rabbit IgG AlexaFluor^®^ 488 (catalogue number ab150077, Abcam), anti-mouse IgG AlexaFluor^®^ 594 (catalogue number ab150116, Abcam) fluorescent secondary antibodies and Hoechst 33342 diluted in PBS for an hour in RT. Finally, the slides were mounted with prolong^®^ gold antifade reagent (catalogue number 9071, cell signaling) and stored, protected from light at 4 °C prior to microscopic observation.

## Results

### Intact mitochondrial function is indispensable for fecundity in *C. elegans*

The mitochondrial genome of *C. elegans* encodes 12 ETC protein-coding genes lacking the ATP8 gene of its human counterpart [20]. The following proteins have been proposed to engage in mtDNA metabolism: HMG-5, a putative nematode homolog to the mammalian TFAM, is a high-mobility group protein that regulates mitochondrial DNA content and has important roles in replication, transcription, and packaging of mtDNA into nucleoids [21, 22]. *C. elegans* genome encodes a TFB1M homolog, a protein with 16S rRNA methyltransferase activity, which shares some properties, but has distinct functions from TFB2M [23]. *Rpom-1* gene encodes the mammalian POLRMT homolog RPOM-1. Alignment of the catalytic domain of RPOM-1 and its putative homologs revealed extensive conservation from yeast to mammals (Fig. S1). We created RNAi constructs to specifically target *hmg-5*, *rpom-1* and *tfbm-1* transcripts (Fig. S2A), since mutants for the respective genes are lethal and sterile. We found that RNAi-mediated knockdown of *hmg-5*, *rpom-1,* and *tfbm-1* compromised mitochondrial function, as evidenced by a decrease in TMRE (Tetramethylrhodamine, ethyl ester, perchlorate) and MitoTracker Red CM-H2XROS stainings, as well as a reduction in ATP levels (Fig. S2B-D). Importantly, RPOM-1 depletion dramatically reduced mtDNA content, at levels comparable to HMG-5 depletion (Fig. S2E). This can be attributed to the fact that POLRMTs also provide RNA primers for the initiation of mtDNA replication by the mitochondrial-specific DNA polymerase. Taken together, these results highlight the importance of intact transcription machinery residing in mitochondria for preserving full organelle function.

**Fig. 1:**
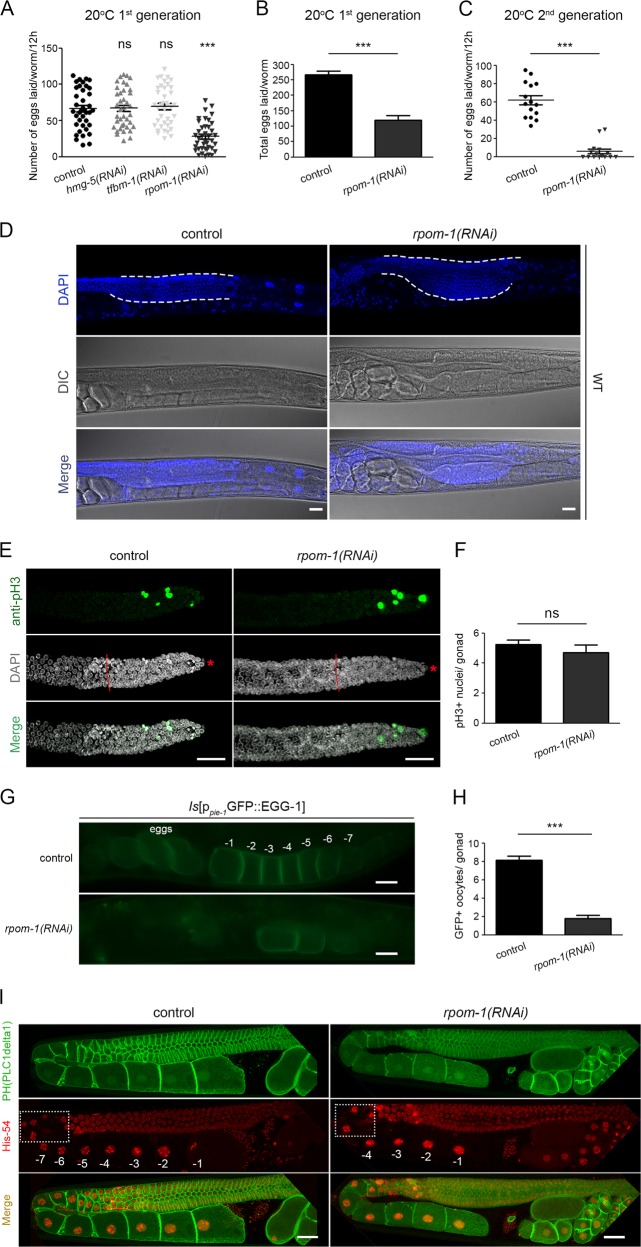
RPOM-1 depletion causes germline tumor formation in *C. elegans*. **a**, **b** The brood size of *rpom-1(RNAi)*-treated hermaphrodites is significantly reduced (up to 50%) compared to their control counterparts. Unpaired *t*-test was used for the estimation of statistical significance (*n* > 40; ***P < 0.001). **c** Egg laying measurement of animals treated with control or *rpom-1(RNAi)* for two subsequent generations. **d** DAPI staining of day 1 control and *rpom-1(RNAi)*-treated WT animals. Inhibition of mitochondrial transcription results in germ nuclei arrest in the pachytene region of prophase I in the germline syncytium. The dashed lines surround the germline syncytium. **e** Phosphorylated histone H3 antibody staining of extruded gonads for the detection of mitotic nuclei in the distal gonad arm. Red dashed lines highlight the border between the mitotic region and the transition zone, marked by the appearance of crescent-shape nuclei, while the red asterisk marks the relative position of the distal gonad arm tip. **f** Quantification of phosphorylated histone H3 positive germ nuclei in control and *rpom-1(RNAi)*-treated hermaphrodites. **g** Representative images of EGG-1 positive oocytes in the proximal arm from control and RPOM-1-depleted gonads. **h** Quantification of EGG-1 positive oocytes in control and *rpom-1(RNAi)*-treated hermaphrodites. **i** Confocal image of day 1 transgenic worms expressing a cell membrane tagged GFP and a histone-54 fused mCherry. Animals treated with *rpom-1(RNAi)* display a lower number of nuclei in diplotene (compare dashed rectangles) and fewer mature oocytes in diakinesis. −1 denotes the most proximal oocyte. Unpaired *t*-test was used for the estimation of statistical significance (*n* > 40; ***P < 0.001). Error bars, s.e.m. Images were acquired using a ×40 objective lens. Scale bars, 20 μm

We noticed that *rpom-1* knockdown dramatically reduced the brood size of wild-type nematodes (Fig. 1a, b). The defect was even more pronounced in subsequent generations, indicating the existence of a maternal-effect phenotype (Fig. 1c). Intriguingly, we detected the formation of germline tumors in the pachytene syncytium region of animals fed with bacteria expressing *rpom-1(RNAi)* (Fig. 1d). We reasoned that this phenotype could be a consequence of elevated mitotic activity in the proliferative region of the gonad. To test this hypothesis, we stained extruded gonads from control and *rpom-1(RNAi)*-treated animals with an antibody specific to phosphorylated serine 10 of histone H3 (anti-pH3), a widely-used marker for mitotic-phase nuclei [24, 25]. RPOM-1 depletion did not cause any significant change in the number of mitotic nuclei (Fig. 1e, f). We then investigated whether germ nuclei accumulate due to their inability to properly differentiate and produce oocytes. By utilizing a fluorescent reporter strain for EGG-1, a protein that localizes to oocyte membranes, we detected three times less EGG-1::GFP positive oocytes in *rpom-1(RNAi)*-treated animals compared to their control counterparts, as well as less nuclei in diplotene and diakinesis (Fig. 1g–i). Furthermore, we did not observe any gross morphological defect in somatic tissues that support the gonads, such as the gonad sheath (Fig. S3Α) and the DTC (Fig. S3Β) or any change in the number of sperm nuclei that the animals produce (Fig. S3C, D). These observations confirm that impaired differentiation of germ cells to oocytes leads to germline tumor development in *rpom-1(RNAi)*-treated animals.

### Germline apoptosis counterbalances tumor formation

**Fig. 2:**
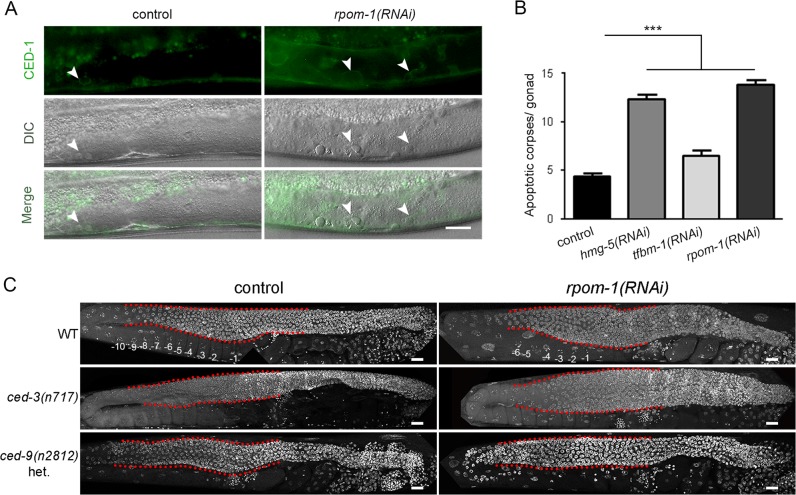
Induction of apoptosis alleviates germline tumor development. **a** Apoptosis induction following *rpom-1* downregulation, as monitored using the CED-1::GFP reporter in combination with DIC microscopy. Arrows highlight apoptotic corpses in the syncytium area. **b** A two-fold induction in the number of early apoptotic corpses can be detected upon RPOM-1 depletion. *Hmg-5* and *tfbm-1* downregulation also trigger apoptosis (*n* = 40; ***P < 0.001, one-way ANOVA was used for multiple comparisons). **c** RPOM-1 depletion in *ced-3(n717)*/caspase-deficient animals causes pronounced tumor formation, even more severe than in wild-type worms. Heterozygous *ced-9(n2812)*/BCL-2 animals exhibit no sign of germ nuclei arrest in pachytene. The red dashed lines surround the pachytene region of the gonads. −1 denotes the most proximal oocyte. Images were acquired using a ×40 objective lens. Error bars, s.e.m. Scale bars, 20 μm

We surveyed for cellular responses triggered by aberrant mitochondrial biogenesis. RPOM-1 depletion resulted in accumulation of apoptotic corpses in the gonad syncytium, which were clearly evident using differential interference contrast (DIC; Nomarski) microscopy and a CED-1::GFP reporter strain (Fig. 2a). CED-1 is a transmembrane receptor which normally clusters around apoptotic corpses before they are engulfed and removed by gonad sheath cells [26, 27]. Furthermore, both HMG-5 and TFBM-1 depletion efficiently induced apoptosis, although to a lesser extent than *rpom-1(RNAi)* (Fig. 2b), indicating that distinct signals emanating from dysfunctional mitochondria may trigger programmed cell death. Inhibition of apoptosis in homozygous *ced-3*/caspase mutants can lead to the accumulation of germ cell corpses that cannot be removed [28]. Notably, *rpom-1* knockdown in *ced-3(n717)* homozygous mutants aggravated the pachytene arrest phenotype observed in wild-type nematodes. By contrast, loss of function mutants for *ced-9(n2814)*/BCL-2, where apoptosis is induced, exhibit no signs of tumor formation upon *rpom-1* downregulation (Fig. 2c). Hence, induction of apoptosis compensates for the tumor phenotype caused by the inhibition of mitochondrial transcription.

### Mitochondrial transcription acts in parallel with signaling pathways converging on the germline

**Fig. 3:**
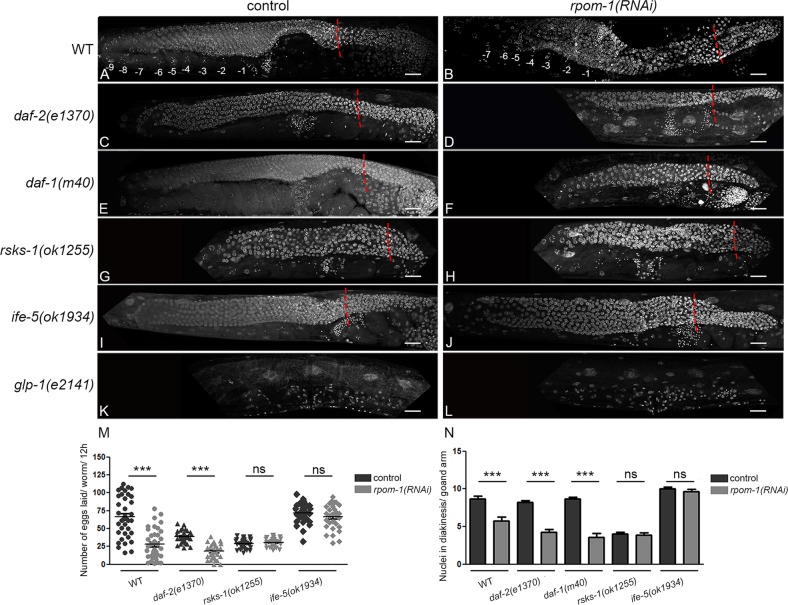
Mitochondrial transcription acts in parallel with signaling pathways converging on the germline. Hoechst 33342 staining of D1 adult animals in control conditions and upon *rpom-1* silencing. **a**, **b** Inhibition of mitochondrial transcription causes pachytene arrest in otherwise wild-type animals. In contrast, treatment of *daf-2*/ IGFR (**c**, **d**) and *daf-1*/ TGFR (**e**, **f**) homozygous mutants with *rpom-1(RNAi)* at 20 °C produces dwarf gonads and augments their reported defects. Mutants with attenuated protein synthesis rates, such as *rsks-1*/ S6K (**g**, **h**) and *ife-5*/eIF4E (**i**, **j**) are indistinguishable from their control counterparts upon treatment with *rpom-1(RNAi)*. **k**, **l** *Glp-1*/ Notch loss of function produces germline-less animals at restrictive temperatures. The red dashed lines indicate the border between the mitotic region and the transition zone. **m** Egg laying measurements in wild-type, *daf-2(e1370)*, *rsks-1(ok1255)* and *ife-5(ok1934)* mutants. **n** Quantification of the number of germ nuclei reaching diakinesis in wild-type, *daf-2(e1370)*, *daf-1(m40)*, *rsks-1(ok1255)* and *ife-5(ok1934)* genetic backgrounds. (*n* > 40; ***P < 0.001, unpaired *t*-test). Error bars, s.e.m. Images were acquired using a ×40 objective lens. Scale bars 20 μm

We next investigated the impact of RPOM-1 depletion in mutants with reported defects in germline homeostasis. Insulin/IGF-1 signaling promotes proliferation of germline stem cells [29]. While *rpom-1* downregulation caused tumor formation in the germline syncytium of wild-type animals (Fig. 3a, b), it differentially affected *daf-2(e1370)*/IGFR mutants by producing dwarf gonads at permissive temperatures (20 °C) and aggravating their proliferative defects (Fig. 3c, d). TGF-β signaling is also reported to affect the balance between mitosis and differentiation in the *C. elegans* germline, in response to environmental cues, such as concentration of dauer pheromone or population density [30]. RPOM-1 depletion in *daf-1(m40)*/TGFR mutants behaved similarly to *daf-2(e1370)*/IGFR mutants, since it produced gonads with further compromised proliferative potential compared to their respective controls (Fig. 3e, f). In sharp contrast the gonads of *rsks-1(ok1255)*/S6K mutants, which exhibit attenuated global protein synthesis, were identical to those of control animals (Fig. 3g, h). Importantly, *ife-5(ok1934)*/eIF4E mutants, similarly to *rsks-1(ok1255)* mutant animals, remained unaffected by RPOM-1 depletion (Fig. 3i, j). Furthermore, the egg-laying defect caused by RPOM-1 deficiency was completely absent in protein synthesis-defective nematodes, as evident by counting the brood size of individual animals and the number of diakinesis-staged nuclei (Fig. 3m, n). This indicates that reduced protein synthesis provides a selective advantage under conditions of impaired mitochondrial ATP production. Finally, *rpom-1(RNAi)*-treated *glp-1(e2141)*/Notch loss of function mutants was indistinguishable from controls under restrictive temperatures, producing germline-less hermaphrodites (Fig. 3k, l). Numerous mutant strains for vital germline components, such as P-granules, are fully fertile at standard growth conditions (20 °C), but become sterile following a switch to a higher temperature (25 °C) [31]. The defects accompanying RPOM-1 depletion became more prominent when the animals were raised at 25 °C. The gonads at that temperature virtually collapse, barely produce a few diplotene and diakinesis-staged germ nuclei and accumulate dead corpses (Fig. S4). Together, these findings suggest that the precise coordination of signaling pathways (Insulin/IGF-1, TGF-β, and Notch/Delta) and internal gonad processes (mitochondrial transcription and protein synthesis) shapes the balance between mitosis and meiosis in the *C. elegans* germline.

### *rpom-1* expression is compartmentalized

**Fig. 4:**
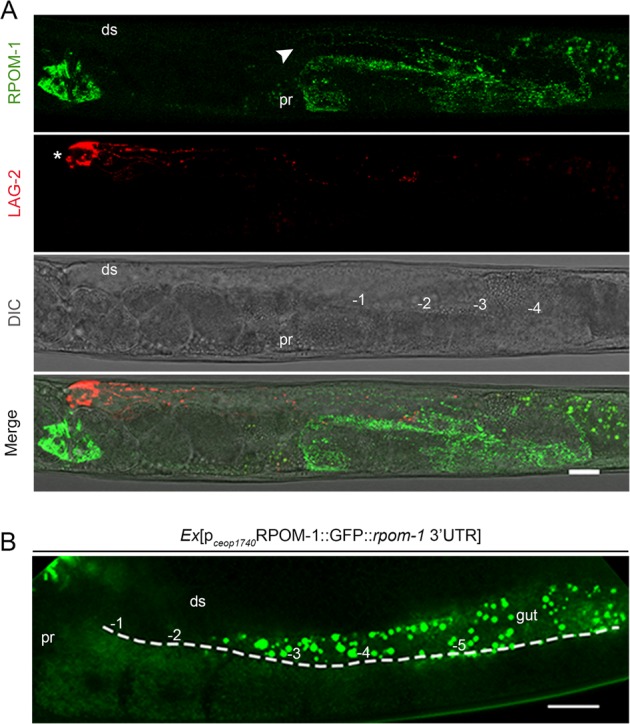
*Rpom-1* expression is compartmentalized. **a** RPOM-1 abundance (in green) is low in the distal arm and the mitotic region of the gonads, becomes evident at the onset of the pachytene region (arrowhead) and substantially increases close to the turn and in the proximal arm, where the oocytes mature. LAG-2::myr::tdTomato (in red) marks the distal tip cell (white star) and its membrane projections. **b** A reporter strain overexpressing RPOM-1 under the control of its endogenous promoter and 3′UTR reveals higher expression in the oocytes and lower in the syncytium. Ds; distal, pr; proximal, −1 denotes the most proximal oocyte. Images were acquired using ×40 and ×63 objective lenses. Scale bars, 20 μm

To monitor *rpom-1* expression in vivo, we generated a translational reporter by fusing GFP to the carboxyl terminus of full-length *rpom-1* cDNA regulated by its endogenous operon promoter. *rpom-1* is expressed in several somatic tissues, including muscles, intestine, vulva, and neuronal cells in the nerve ring and the tail, in a pattern reminiscent of proteins localizing in the mitochondrial matrix (Fig. S5). *rpom-1* is also strongly expressed in the germline of hermaphrodite animals in a punctuate pattern. In the gonads, mitochondria surround and enwrap germ cell nuclei in the syncytium region (Fig. S6A, B). We observed a clear colocalization of RPOM-1 with TMRE, a potential-dependent mitochondrial dye (Fig. S6C). We crossed RPOM-1::GFP transgenic animals with p_*lag-2*_MYR::tdTomato reporter animals, to visualize RPOM-1 expression in regard to the position of the DTC. We noticed that *rpom-1* expression is low in the mitotic region distally, but increases profoundly as germ cell nuclei mature to form oocytes (Fig. 4a, Fig. S7A). Interestingly, RPOM-1 expression increases at the onset of pachytene, the exact same region where germ nuclei arrest upon RPOM-1 depletion. To achieve a faithful reconstitution of the endogenous *rpom-1* expression pattern, we also generated a reporter strain that expresses a transgene carrying the endogenous *rpom-1* 3′UTR. On a similar note, RPOM-1 was enriched in the oocytes of the proximal gonad arm (Fig. 4b). The expression of the operon’s promoter per se gradually increases in a distal to proximal manner (Fig. S7B). IFET-1, which localizes to P-granules and is required for normal gonad development, is a general translational repressor in the germline [32]. Treatment of our reporter animals with *ifet-1(RNAi)* de-repressed *rpom-1* expression distally (Fig. S7C). Hence, *rpom-1* expression in the germline is compartmentalized, increases as germ nuclei progress to the proximal arm and form oocytes and is directly regulated by IFET-1 at the translational level.

### Transition to tubular mitochondria is a hallmark of differentiation

**Fig. 5:**
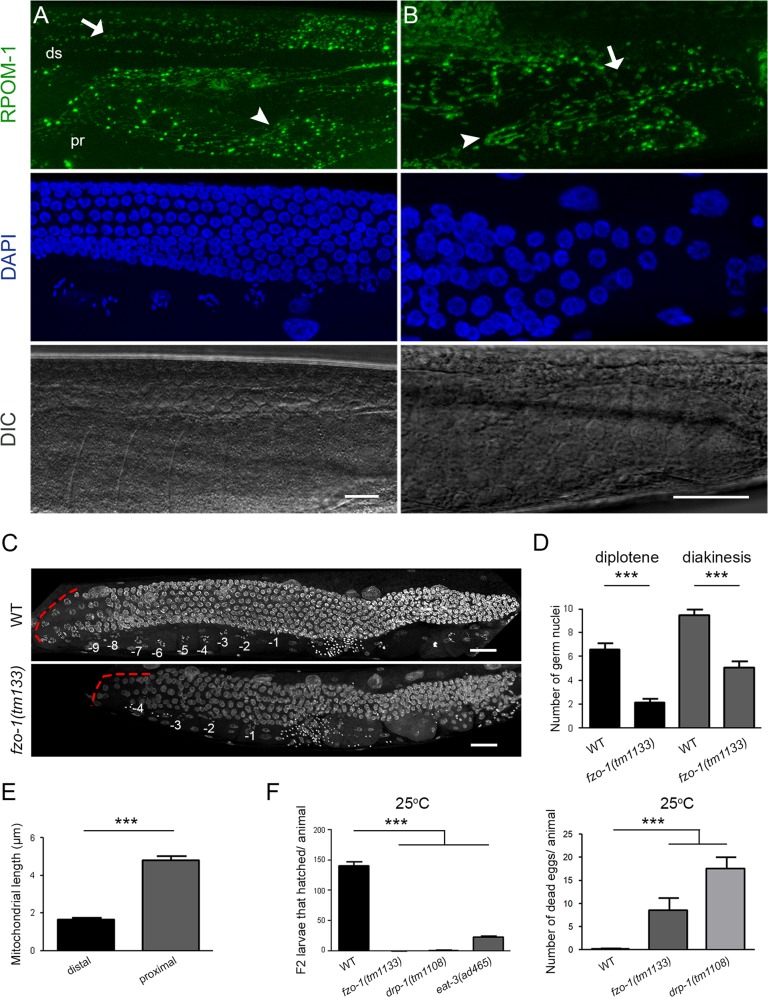
Transition from globular to tubular mitochondria is a prerequisite for germline homeostasis. **a** Confocal image of an adult *C. elegans* gonad. In the distal arm, mitochondria have a globular shape (arrow), which gradually switches to a more elongated/ tubular one in the oocytes of the proximal arm (arrowhead). **b** The turn of the gonad, shown in magnification, is the site where the shape alteration occurs. There, both globular (arrow) and tubular (arrowhead) mitochondria can be observed. **c** DAPI staining of wild-type and *fzo-1(tm1133)*/Mitofusin homozygous mutants. The red dashed line marks the gonad turn. **d** Day 1 adult *fzo-1(tm1133)*/Mitofusin mutant animals, bearing defects in mitochondrial fusion, produce significantly fewer germ nuclei in diplotene as well as nuclei in diakinesis compared to their control counterparts. (*n* > 40; ***P < 0.001, unpaired *t*-test). **e** Quantification of mitochondrial length in the proximal and distal arm of the gonads. **f** Animals with perturbed mitochondrial dynamics (fusion-fission), such as *fzo-1*, *drp-1* and *eat-3* mutants become sterile when exposed to a mild heat stress (25 °C). One-way ANOVA was used for the estimation of statistical significance (*n* > 40; ***P < 0.001). Error bars, s.e.m. Images were acquired using X40 and X63 objective lenses. Ds; distal, pr; proximal, −1 denotes the proximal-most oocyte. Scale bars, 20 μm

We noticed that mitochondrial morphology alters in the course of germ cell differentiation. The distal gonad arm was abundant with globular mitochondria, while elongated organelles prevailed in the proximal arm (Fig. 5a, e). In the gonad turn both globular and tubular mitochondria coexist (Fig. 5b). In that region, an actin-dependent cytoplasmic streaming deposits cytoplasmic material and mitochondria to the oocytes [33]. Accumulating evidence suggests that elongated mitochondria are associated with elevated ETC activity and vice versa [34, 35]. Mitochondrial shape is malleable and alters to fulfill physiological demands, in response to stress and other intracellular or environmental signals. Specialized dynamin GTPases FZO-1/Mitofusin and EAT-3/OPA-1 mediate fusion, while DRP-1 is required for fission [36]. Interestingly, *fzo-1(tm1133)* mutant animals possess significantly less germ nuclei in diplotene as well as oocytes in diakinesis, producing fewer offspring (Fig. 5c, d). Furthermore, while *fzo-1(tm1133)* and *drp-1(tm1108)* mutants were viable and fertile at 20^o^C, they became sterile when the rearing temperature was shifted to 25^o^C, producing mainly dead eggs and negligible offspring (Fig. 5f). Together, these findings support the notion that tubular mitochondria in the proximal arm represent the outcome of a maturation process, which is essential for oocytes to cope with their high-energy demands.

**Fig. 6:**
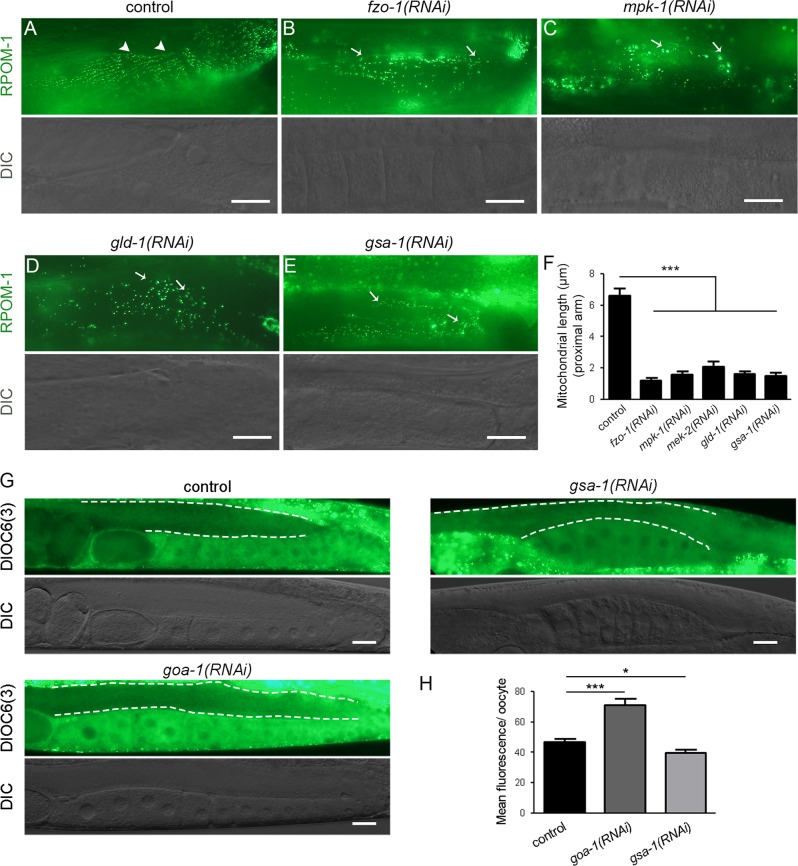
Sperm-derived signals promote mitochondrial maturation. **a** Mitochondria in the proximal gonad arm are tubular under control conditions (arrowheads). **b** Knockdown of *fzo-1* leads to mitochondrial network fragmentation and globular mitochondria in the proximal gonad arm (arrows). **c** Inhibition of MPK-1/MAPK signaling via *mpk-1(RNAi)* results in a failure of mitochondria to elongate proximally (arrows). **d** Mitochondria are exclusively globular in the proximal arm of *gld-1(RNAi)*-treated animals (arrows). **e** GSA-1 inhibition results in failure of oocyte maturation as well as mitochondrial elongation. **f** Quantification of mitochondrial length in proximal gonad arm oocytes upon the respective RNAi treatments (*n* = 40; ***P < 0.001, one-way ANOVA was used for multiple comparisons). **g** DIOC6(3) staining reveals that mitochondria polarize in the course of germ cell differentiation. Inhibition of GOA-1, a negative regulator of sperm signaling, boosts mitochondrial potential in the proximal gonad arm. Treatment with *gsa-1(RNAi)* results in a failure of mitochondria to polarize proximally. **h** Quantification of the DIOC6(3) fluorescence per oocyte of the proximal gonad arm. The dashed lines surround the germline syncytium. (*n* = 40; ***P < 0.001, unpaired *t*-test). Error bars, s.e.m. Images were acquired using a X40 objective lens. Scale bar, 20 μm

To shed light on the molecular mechanism that governs the alteration of mitochondrial morphology, we focused on well characterized pathways associated with germ cell differentiation in *C. elegans*. Spatial activation of MPK-1/MAPK signaling is crucial for germ cell exit from pachytene. MPK-1 deficient mutants exhibit pachytene arrest, phenocopying RPOM-1 depletion [37]. Interestingly, tubular organelles were absent upon knockdown of *mek-2* or *mpk-1*, the homologs of mammalian MEK and ERK kinases, respectively (Fig. 6a, c, f). GLD-1 is an RNA-binding protein that binds to the 3′UTR of target mRNAs, repressing their translation. In *gld-1* loss of function mutants germ nuclei exit meiosis and return to mitosis, forming germline tumors [38]. Similarly to FZO-1/Mitofusin depleted animals, *gld-1(RNAi)*-treated animals contain exclusively globular mitochondria in the proximal gonad arm (Fig. 6a, b, f). Gonad sheath cells are known to respond to sperm-derived signals (major sperm proteins) and promote oocyte maturation through activation of the Gα_s_-adenylate cyclase-protein kinase A pathway [39]. Inhibition of *gsa-1*, the worm Gs alpha subunit of heterotrimeric G proteins, prevents oocyte maturation [40]. Interestingly, mitochondria failed to elongate in the proximal arm of *gsa-1(RNAi)*-treated animals (Fig. 6a, e, f) and the existing organelles were not efficiently polarized (Fig. 6g, h). Conversely, inhibition of GOA-1, a negative regulator of oocyte maturation, boosted mitochondrial potential in the proximal arm (Fig. 6g, h). Altogether, these findings suggest that germ nuclei differentiation is intertwined with mitochondrial maturation and the latter appears to rely on two important signaling pathways, namely MPK-1/MAPK and MSP.

### Mitochondria functionally mature *en route* to germ cell differentiation

**Fig. 7:**
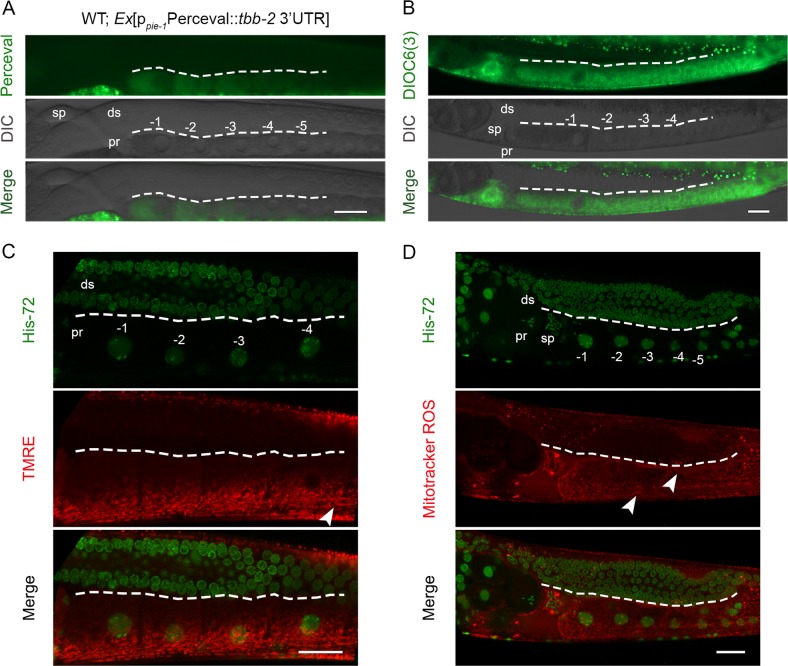
Mitochondria functionally mature during germ nuclei differentiation. **a** The ATP/ADP sensor Perceval was overexpressed in the *C. elegans* germline under the control of *pie-1* promoter, to achieve germline-specific expression. Perceval emission increases upon ATP binding. Fluorescence could be mainly detected in the oocytes, indicating increased ATP production in the proximal arm. **b** DIOC6(3) mitochondrial dye preferentially stains energized mitochondria in the proximal gonad arm. **c** TMRE staining reveals increased electrochemical potential in the oocytes of the proximal arm. **d** Mitochondrial ROS production increases as the germ nuclei mature and give rise to oocytes. Arrowheads highlight tubular mitochondria in the proximal gonad arm. Sp; spermatheca, ds; distal, pr; proximal, −1 denotes the most proximal oocyte. Images were acquired using ×40 and ×63 objective lenses. Scale bars, 20 μm

The established interplay between mitochondrial morphology and metabolic activity [34] prompted us to test mitochondrial maturation within the germline. We cloned Perceval, a fluorescent sensor for adenylate nucleotides [41], downstream of the *pie-1* promoter, to uniformly express it in the germline. We also utilized the *tbb-2* 3′UTR to avoid undesired silencing of our transgene [42]. Interestingly, we detected expression in the oocytes, but not in the gonad syncytium (Fig. 7a). This is consistent with the notion that ATP is produced proximally, in the area that is abundant with tubular mitochondria. Treatment with Antimycin A, a bacterial toxin that inhibits ETC complex III, dampened fluorescence in oocytes and fertilized eggs, proving that Perceval can indeed detect changes in mitochondrial ATP production (Fig. S8). To further verify the previous finding, we stained whole animals with dyes that stain mitochondria in a membrane potential-dependent manner, such as TMRE, DIOC6(3) (3,3′-Dihexyloxacarbocyanine Iodide), and MitoTracker Red CM-H2XROS. In congruence with Perceval findings, mitochondrial membrane potential and ROS levels were elevated in developing oocytes (Fig. 7b–d). Interestingly, *rpom-1* knockdown inhibited mitochondrial polarization in the proximal gonad arm as indicated by DIOC6(3) staining (Fig. S9). We postulate that mitochondrial elongation is a primary step in a maturation process, which results in enhanced mitochondrial polarization and concomitant ATP and ROS production in oocytes.

### Elevated POLRMT expression and switch to tubular mitochondria are conserved during evolution

**Fig. 8:**
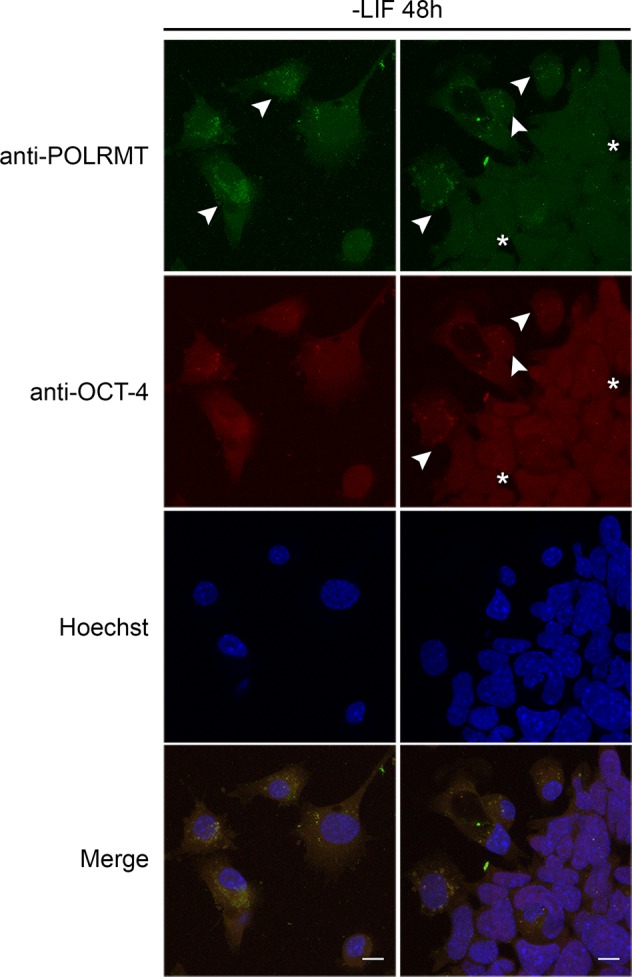
Mammalian stem cell differentiation upon LIF removal is accompanied by an increase in POLRMT expression. Elevated POLRMT expression is observed in cells with increased cytoplasmic OCT-4 abundance (arrowheads). In contrast, adjacent areas with increased nuclear OCT-4 abundance (stars) display lower POLRMT expression. Images were acquired using a ×40 objective lens. Scale bars, 20 μm

We next wondered whether a similar mechanism controls mouse stem cell differentiation. We employed J1 cells, derived from the inner cell mass of mouse blastocysts and grown on spherical colonies in the presence of LIF cytokine. We removed LIF from our culture medium and let the cells differentiate in an unbiased manner. We stained with antibodies for POLRMT and MTCO1, the cytochrome c oxidase subunit I encoded by the mitochondrial genome. The staining was much weaker at the core of the stem cell colonies and progressively increased as cells differentiated and extended membrane projections typical of differentiated cells. Furthermore, 48 h after LIF removal, we observed POLRMT-positive tubular mitochondria (Fig. S10). We also simultaneously stained with POLRMT and OCT-4 antibodies. OCT-4 is a key pluripotency transcription factor that shuttles between the nucleus and the cytoplasm. Its nuclear retention enhances reprogramming efficiency and is associated with pluripotency [43]. Notably, OCT-4 retention in the nucleus was associated with reduced POLRMT expression, while cytoplasmic OCT-4 coincided with increased POLRMT expression (Fig. 8). Collectively, and in line with the *C. elegans* findings, mouse stem cells exhibit low POLRMT expression, while differentiation is accompanied by an increase in POLRMT expression and the appearance of elongated mitochondria.

## Discussion

**Fig. 9:**
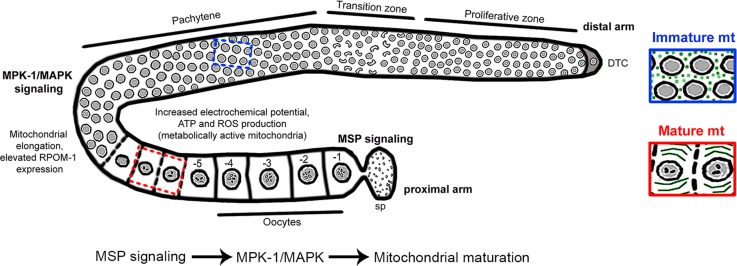
Intact mitochondrial bioenergetics safeguards germline homeostasis. Expression of *rpom-1* (the nematode orthologue of POLRMT) increases progressively as the germ nuclei mature and mitochondria acquire an elongated, tubular shape. The boost in mitochondrial metabolic activity is manifested by enhanced ATP and ROS production, as well as increased electrochemical potential in the proximal gonad arm. Mitochondrial maturation is under the control of MPK-1/MAPK and MSP signaling pathways

The term stem cell niche refers to the specific microenvironment, which ensures that stem cells are protected from harmful agents, divide and differentiate to constantly replenish organs [44]. Each *C. elegans* gonad hosts a unique stem cell niche in an otherwise postmitotic organism [45]. This is the main tissue where mitochondrial DNA replication occurs [46]. A previous study showed that mutation of a germline-specific mitochondrial ATPase subunit impairs fecundity [47]. We find that perturbation of mitochondrial biogenesis, energy production, and dynamics, collectively referred to as bioenergetics, can profoundly affect germ cell differentiation. We describe a maturation process, whereby globular, immature mitochondria are gradually converted to elongated, functional organelles to support increased oocyte energy demands (Fig. 9). This switch is tightly regulated by two core signaling pathways associated with oocyte production and maturation, namely MAPK/ERK and MSP. The MAPK/ERK pathway has pleiotropic functions in the *C. elegans* germline [37]. The DDX-19 helicase and GSK-3 kinase have been shown to be direct MPK-1 targets in vivo [48]. In addition, MPK-1 phosphorylates NOS-3, promoting degradation of TRA-1 by the FEM-CUL2 complex, to facilitate oocyte membrane organization [49]. MPK-1 phosphorylation itself is positively regulated by MSP signaling [50]. Thus, mitochondrial bioenergetics is likely a nodal point modulated by these cascades, in stem cells.

Our findings indicate that expression of mitochondrial RNA polymerase progressively increases during germ cell differentiation. This is accompanied by mitochondrial elongation and manifestation of several hallmarks of metabolic activity, such as increased electrochemical potential, ATP, and ROS production. *Rpom-1* mRNA is one of the numerous targets of FBF-1, a Pumilio family, RNA-binding protein that negatively regulates the expression of mRNAs implicated in meiotic entry [51, 52]. In addition, localized transcription and mRNA translation likely contribute to the subcellular compartmentalization of RPOM-1. Taken together, our observations indicate that upon perturbation of germ cell bioenergetics, germ nuclei stall in the pachytene stage and fail to differentiate, generating fewer oocytes. Consistent with this notion, mitochondrial ATP synthase function is required for the maturation of mitochondrial cristae in *Drosophila* ovaries [53]. Similarly, the pluripotent state of mammalian stem cells has been linked to decreased mitochondrial respiration, in favor of anaerobic glycolysis [54]. Previous studies have demonstrated that mitochondrial mass, mtDNA copy number, and oxygen consumption increase during stem cell differentiation [55–57]. By contrast, successful induction of pluripotent stem cell (iPSC) lines is marked by a reduction of ETC function [58]. Hence, a switch to enhanced mitochondrial respiration is a prerequisite for stem cell differentiation across species.

Conserved signaling pathways implicated in lifespan regulation and dauer formation, also influence germline homeostasis. For instance, Insulin/IGF-1 promotes germ cell proliferation, while DAF-16/FOXO is beneficial for stem cell pool maintenance during ageing [29, 59]. Furthermore, TORC1 and RSKS-1/S6K are required for efficient proliferation of germ cell progenitors [60]. In addition, ASI neuron-derived TGF-β signals determine the balance between mitosis and differentiation in the *C. elegans* germline [30]. Attenuation of Insulin/IGF-1 and TGF-β signaling, combined with perturbation of mitochondrial transcription generates an atrophic germline, and exacerbates proliferation defects, indicating that mitochondria act in concert with extrinsic growth stimuli to dictate mitosis versus differentiation decisions. Similarly, energy is diverted to stress resistance and maintenance mechanisms in mutants with reduced protein synthesis [61, 62]. In this context, as the disposable soma concept postulates, damage repair takes precedence over protein synthesis for germline maintenance and reproduction.

The precise balance between mitosis and differentiation is of utmost importance for tissue and organismal homeostasis. Our work provides novel insights on how mitochondrial bioenergetics dictates cell fate decisions and integrates mitochondria at the core of the developmental modules that shape the *C. elegans* germline. Perturbation of mitochondrial function obstructs germ nuclei differentiation and causes cancer-like phenotypes. A main challenge for future research is to delineate the molecular underpinnings of the germline mitochondrial metabolic switch as well as its temporal and spatial regulation.

## Supplementary information

Supplementary material
