# Supplementary material for: Mitochondrial maturation drives germline stem cell differentiation in Caenorhabditiselegans
Source: Cell Death Differ. 2019 Jun 19;27(2):601–17. doi: 10.1038/s41418-019-0375-9 (PMC7206027; doi:10.1038/s41418-019-0375-9)
Supplement: Supplementary file 1 — Supplementary material [file 41418_2019_375_MOESM1_ESM.docx]

**Mitochondrial maturation drives germline stem cell differentiation**

**in *Caenorhabditis elegans***

Nikolaos Charmpilas and Nektarios Tavernarakis

**SUPPORTING INFORMATION**

**
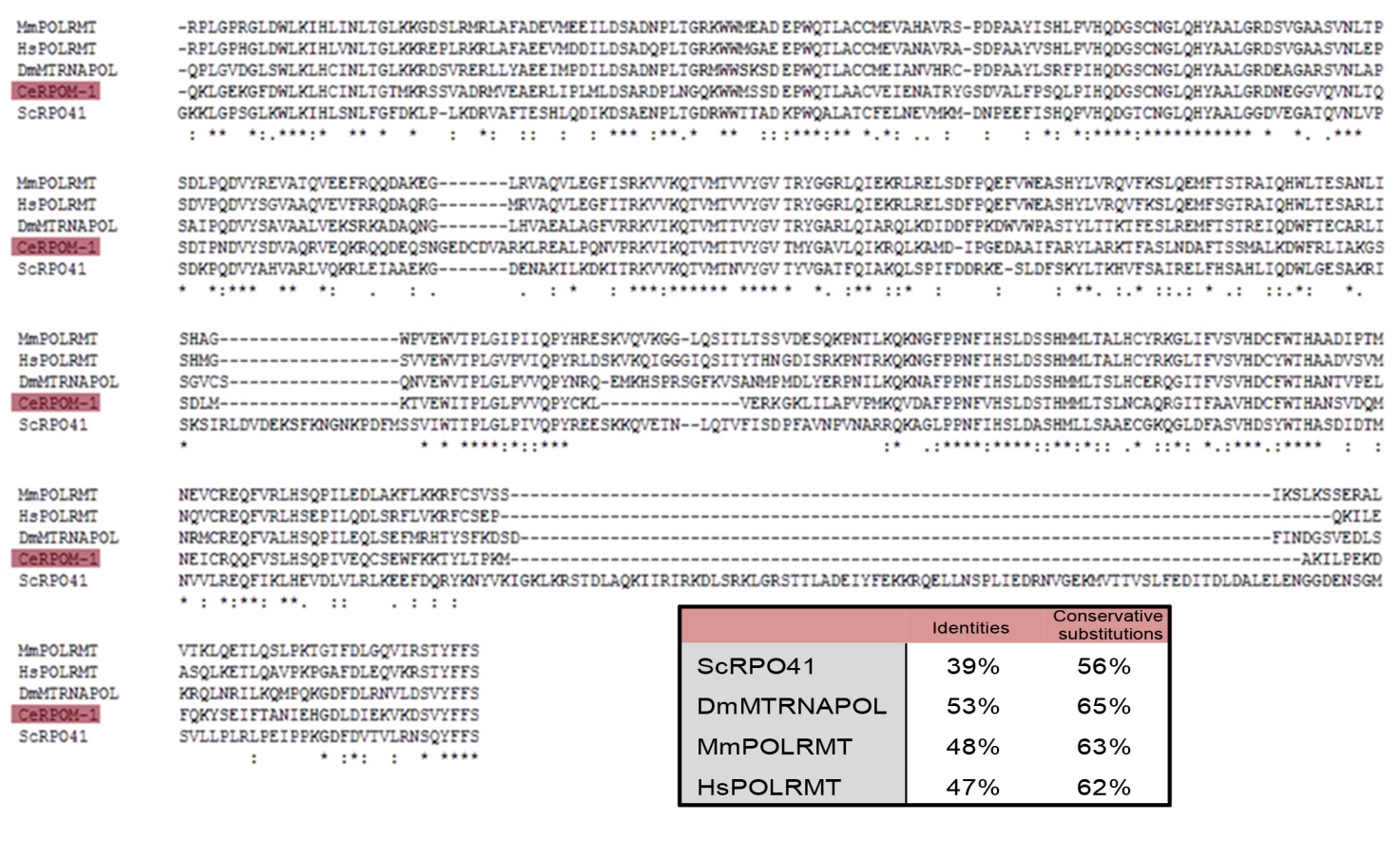
**

**Figure S1: The catalytic domain of mitochondrial RNA polymerases is highly conserved.** CLUSTALW alignment of the catalytic domains from five model organisms (*S. cerevisiae*, *D. melanogaster*, *M. musculus*, *H. sapiens*) reveals high conservation of POLRMTs in evolutionarily diverse species. The table displays the percentage of identical residues as well as conservative substitutions in pair-wise comparisons of *C. elegans* mitochondrial RNA polymerase (RPOM-1) and its identified homologues.


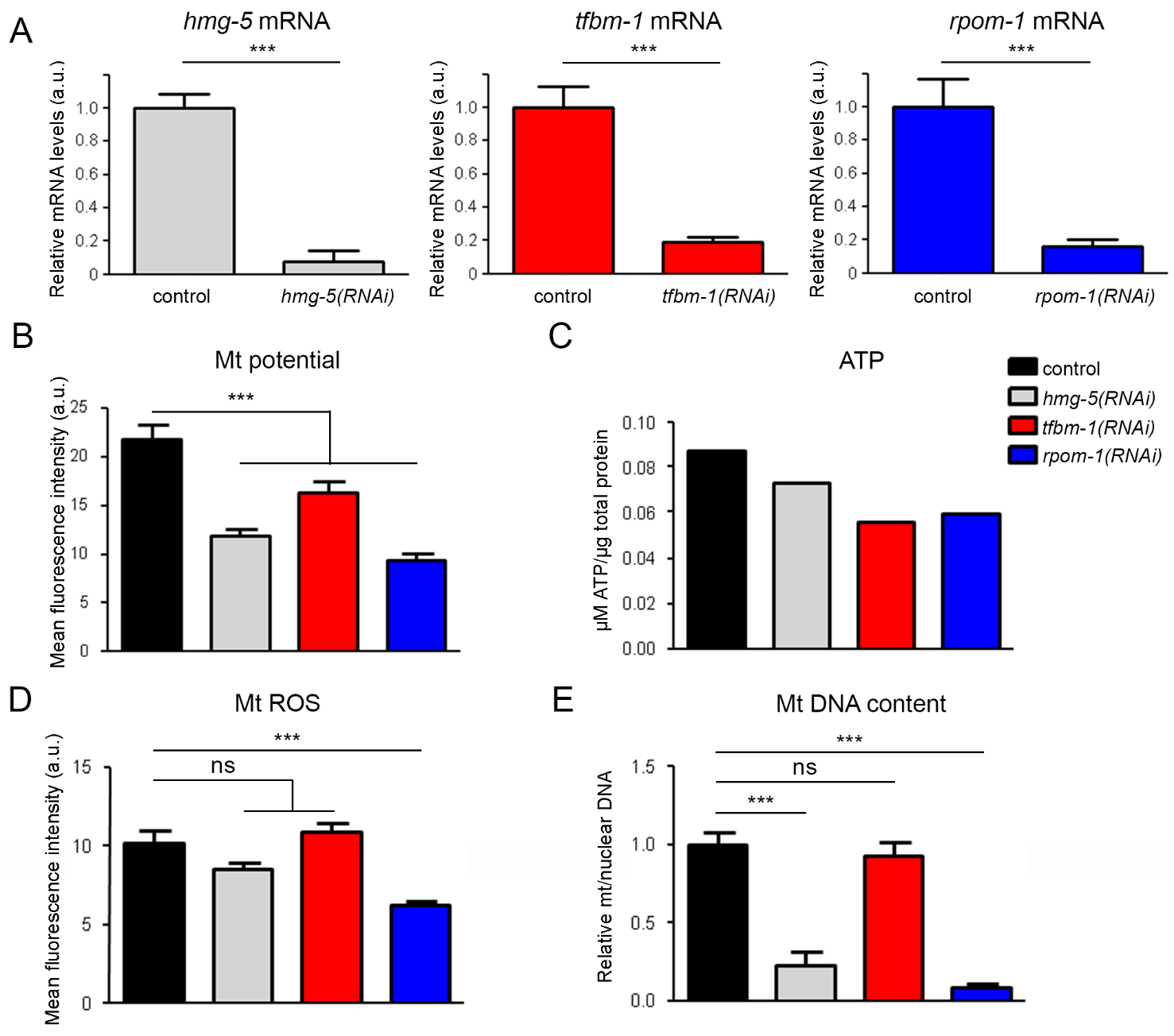


**Figure S2: Reduction of mitochondrial transcription compromises mitochondrial activity.** A) Quantification of *hmg-5*, *tfbm-1* and *rpom-1* mRNA levels upon feeding with RNAi expressing bacteria. Mitochondrial function following genetic inhibition of *hmg-5*/TFAM, *rpom-1*/POLRMT and *tfbm-1*/TFB1M was assayed by staining with potential-based dyes, such as TMRE (B), Mitotracker ROS (D) and by measuring ATP production (C). E) RPOM-1 depletion reduces mitochondrial DNA content, at levels comparable to HMG-5 depletion. One-way ANOVA was used for multiple comparisons (n=40; ****P* < 0.001). Unpaired *t*-test was used for pairwise comparisons (n=40; ****P* < 0.001). Error bars, s.e.m.

**
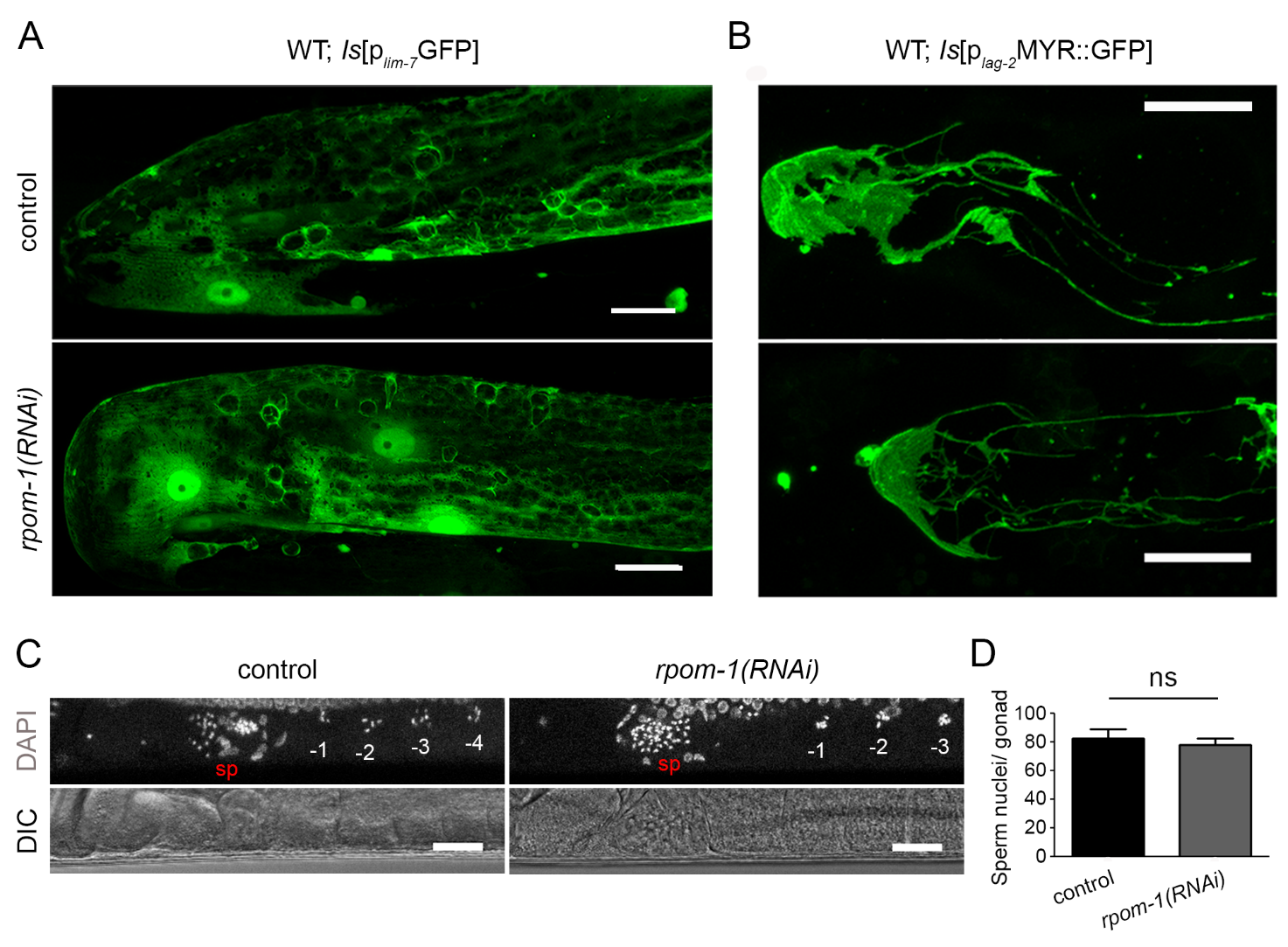
**

**Figure S3: The somatic gonad and sperm number are not affected by RPOM-1 depletion.** The gonad sheath and the distal tip cell do not display any observable morphological defect following RPOM-1 depletion, as indicated by the p*_lim-7_*GFP (A) and p*_lag-2_*MYR::GFP (B) transcriptional reporters, respectively. C) Confocal images of the proximal gonad arm upon treatment with control and *rpom-1(RNAi)*. sp stands for sperm and -1 denotes the most proximal oocyte. D) Quantification of sperm nuclei per gonad in day 2 adult animals upon treatment with control and *rpom-1(RNAi)*. Images were acquired using a X40 objective lens. Scale bars, 20μm.


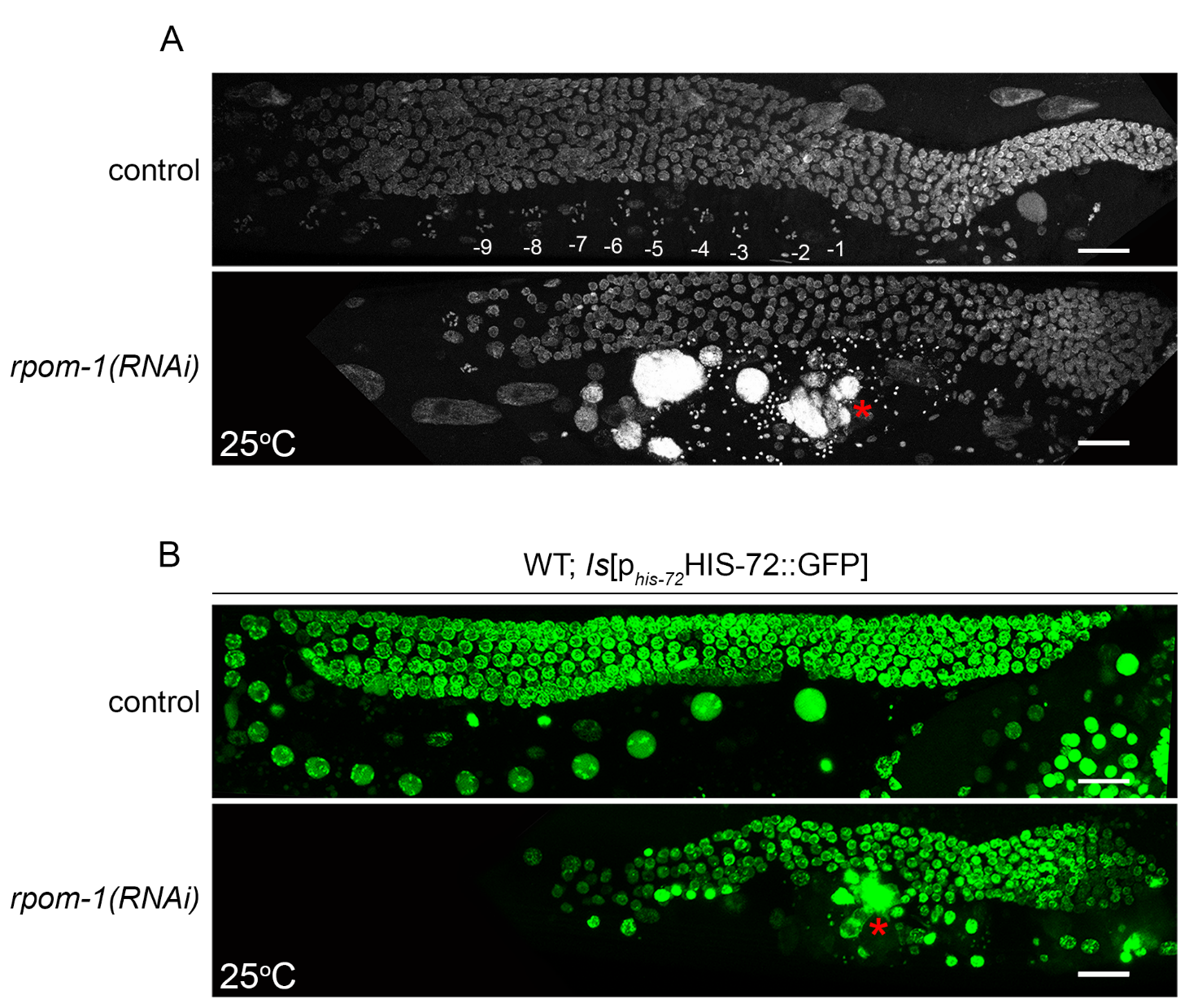


**Figure S4: RPOM-1-depleted gonads are sensitive to mild heat stress.** *rpom-1* knockdown in animals subjected to mild heat stress(25^o^C) is detrimental for gonads, as monitored with DAPI staining in fixed animals (A) and with a HIS-72::GFP nuclear reporter *in vivo* (B). The red stars highlight dead corpses in the proximal gonad arm. Images were acquired using a X40 objective lens. Scale bars, 20μm.


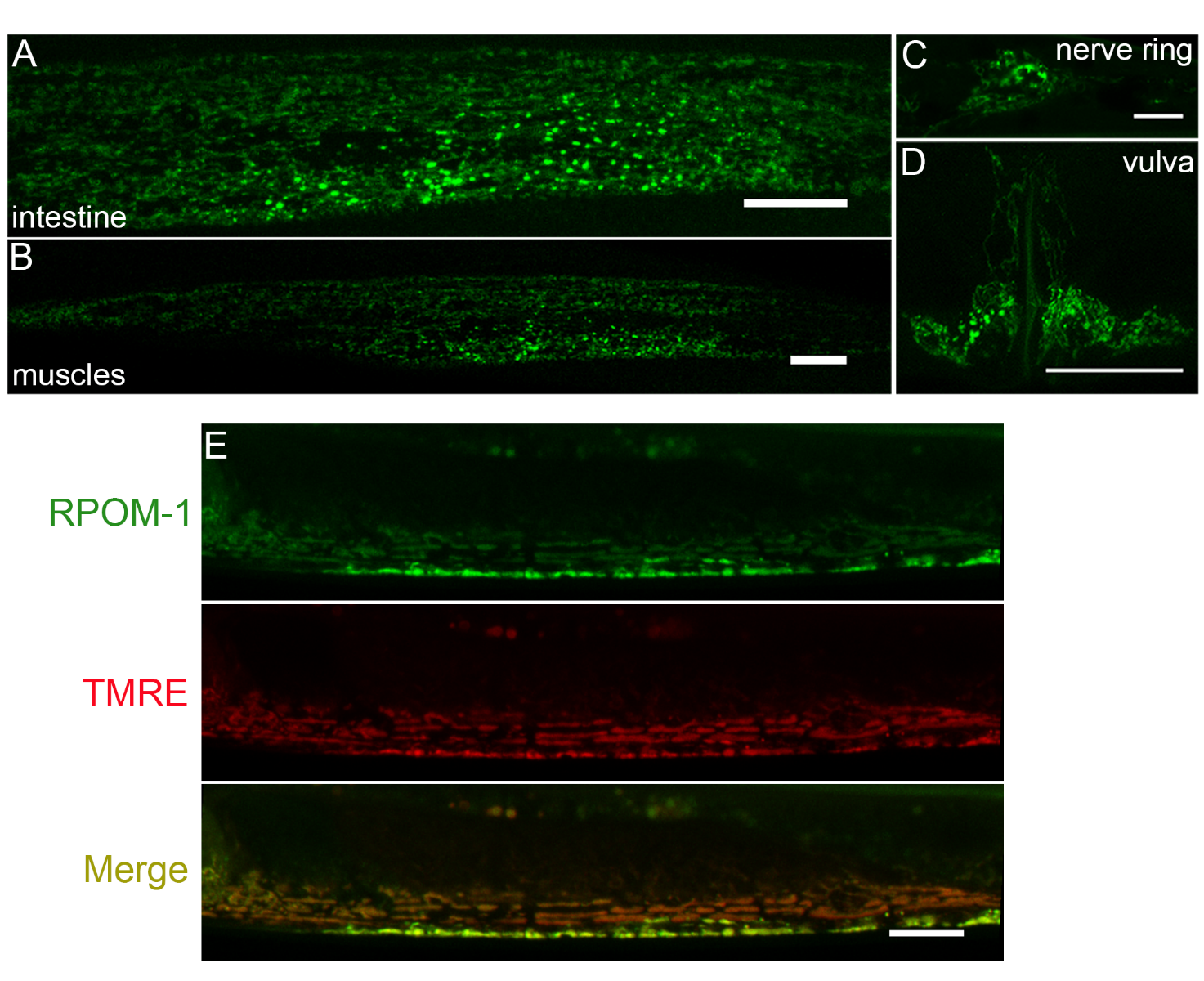


**Figure S5: RPOM-1 is broadly expressed in *C. elegans* somatic tissues and localizes in mitochondria.** RPOM-1 is expressed in various somatic tissues, such as the intestine (A), muscles (B), in neurons of the nerve ring (C), as well as the vulva (D). E) Confocal image of RPOM-1::GFP translational reporter animals stained with TMRE (Tetramethylrhodamine, ethyl ester, perchlorate). The expression pattern is reminiscent of proteins localized in the mitochondrial matrix. Images were acquired using a X40 objective lens. Scale bars, 20μm.


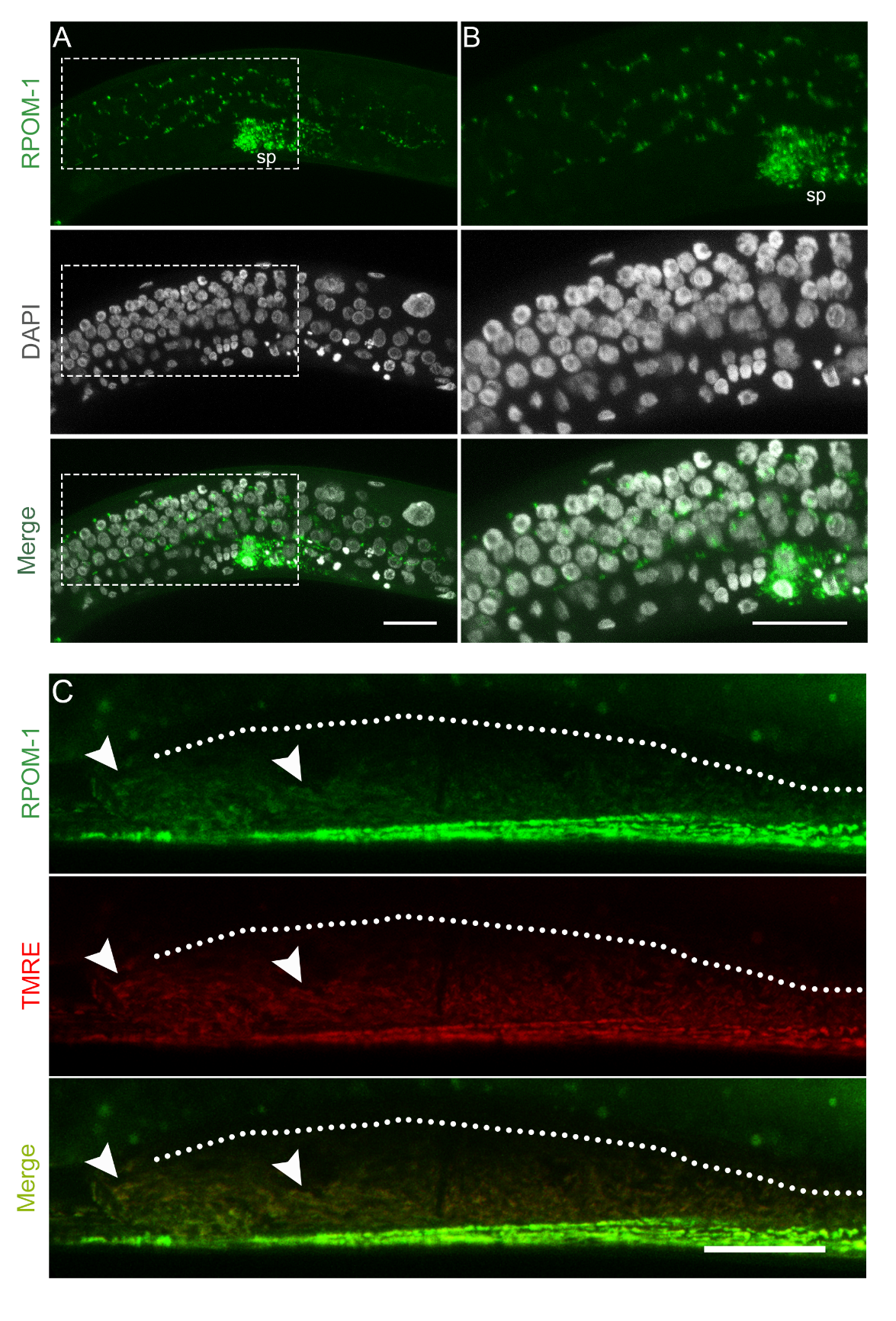


**Figure S6: RPOM-1 expression in the germline.** A) DAPI staining of transgenic animals harbouring an RPOM-1 translational reporter, at late L4 stage, reveals syncytium mitochondria which enwrap germline nuclei. B) Zoom-in of the highlighted area shows high RPOM-1 expression in the spermatheca (sp). C) Staining of RPOM-1::GFP translational reporter animals with TMRE (Tetramethylrhodamine, ethyl ester, perchlorate). Arrowheads indicate tubular mitochondria in the proximal gonad arm, where the two signals co-localize. Images were acquired using X40 and X63 objective lenses. Scale bars, 20μm.


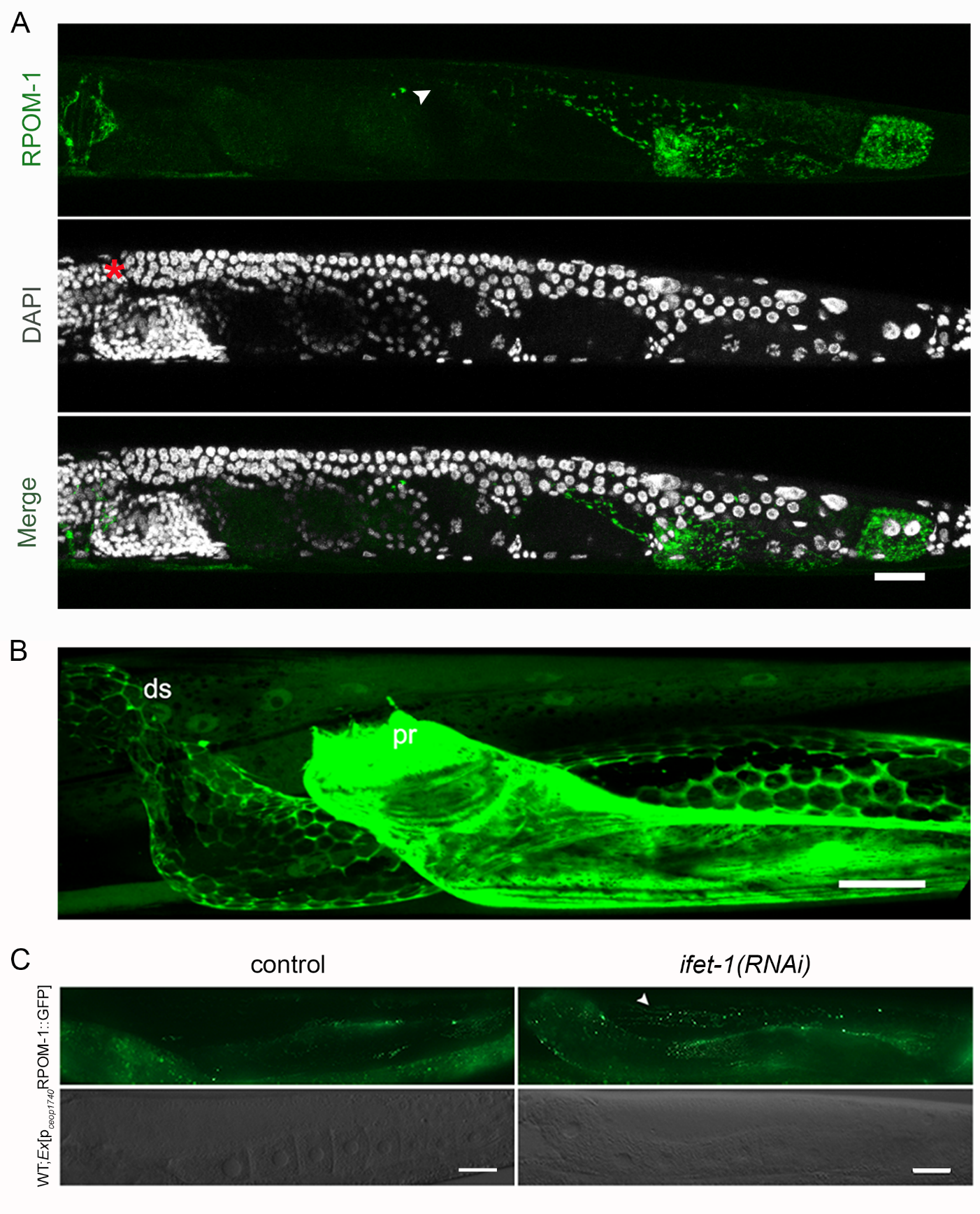


**Figure S7: RPOM-1 accumulates in differentiated germ cells and oocytes.** A) Image of a full gonad from a D1 adult RPOM-1::GFP transgenic worm stained with DAPI. The arrowheads point to the distal-most expression observed, while the red asterisk marks the relative position of the distal tip cell. B) Gonadal expression of a p*_ceop1740_*GFP transcriptional reporter. C) *ifet-1* knockdown de-represses RPOM-1 expression in more distal regions of the gonad syncytium. Images were acquired using a X40 objective lens. Ds; distal, pr; proximal. Scale bars, 20μm.


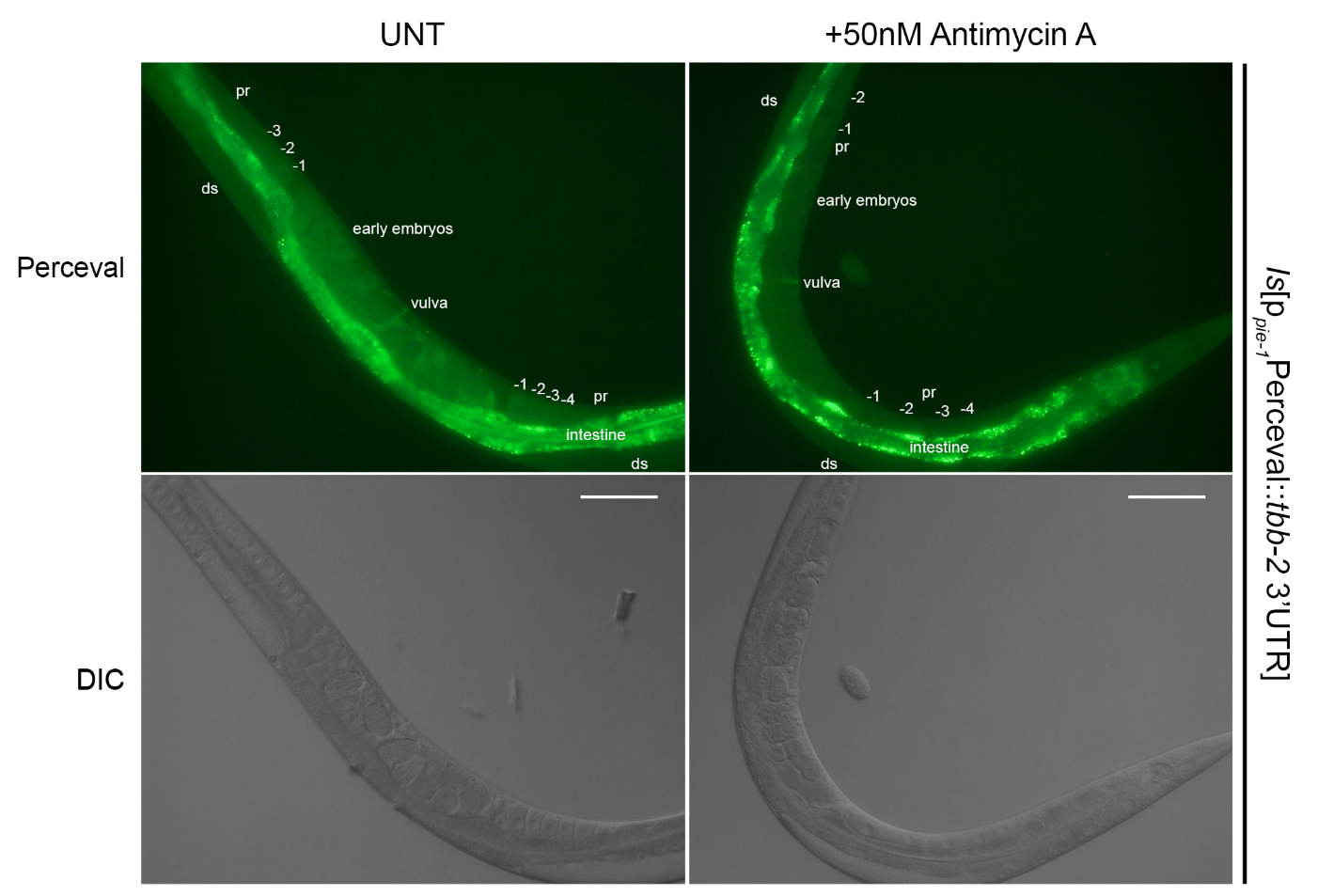


**Figure S8: The Perceval reporter responds to changes in ATP production.** Representative, low-magnification images of transgenic animals expressing the Perceval ATP sensor at D3 of adulthood. Perceval is expressed in the oocytes located in proximal gonad arm, as well as in early embryos. Treatment for two days with 50nM Antimycin A, an inhibitor of ETC complex III and mitochondrial ATP production, reduces the fluorescence the Perceval sensor emits. Images were acquired using a X20 objective lens. -1 denotes the most proximal oocyte. Ds; distal, pr; proximal. Scale bars, 100μm.


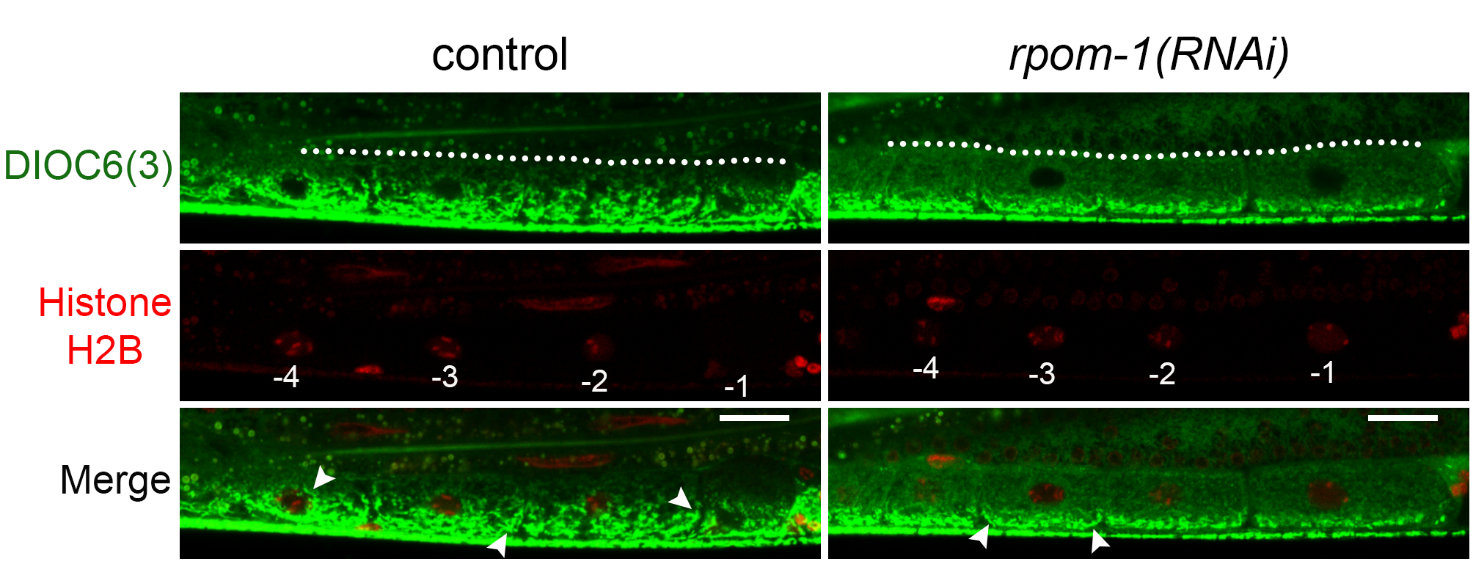


**Figure S9: RPOM-1 is required for mitochondrial maturation in the proximal gonad arm.** DIOC6(3) staining of D1 adult hermaphrodites treated with control or *rpom-1(RNAi)* from hatching. Arrowheads highlight tubular mitochondria in the proximal gonad arm. The white dashed lines indicate dorsal oocyte membranes. -1 denotes the most proximal oocyte. Images were acquired using a X40 objective lens. Scale bars, 20μm.


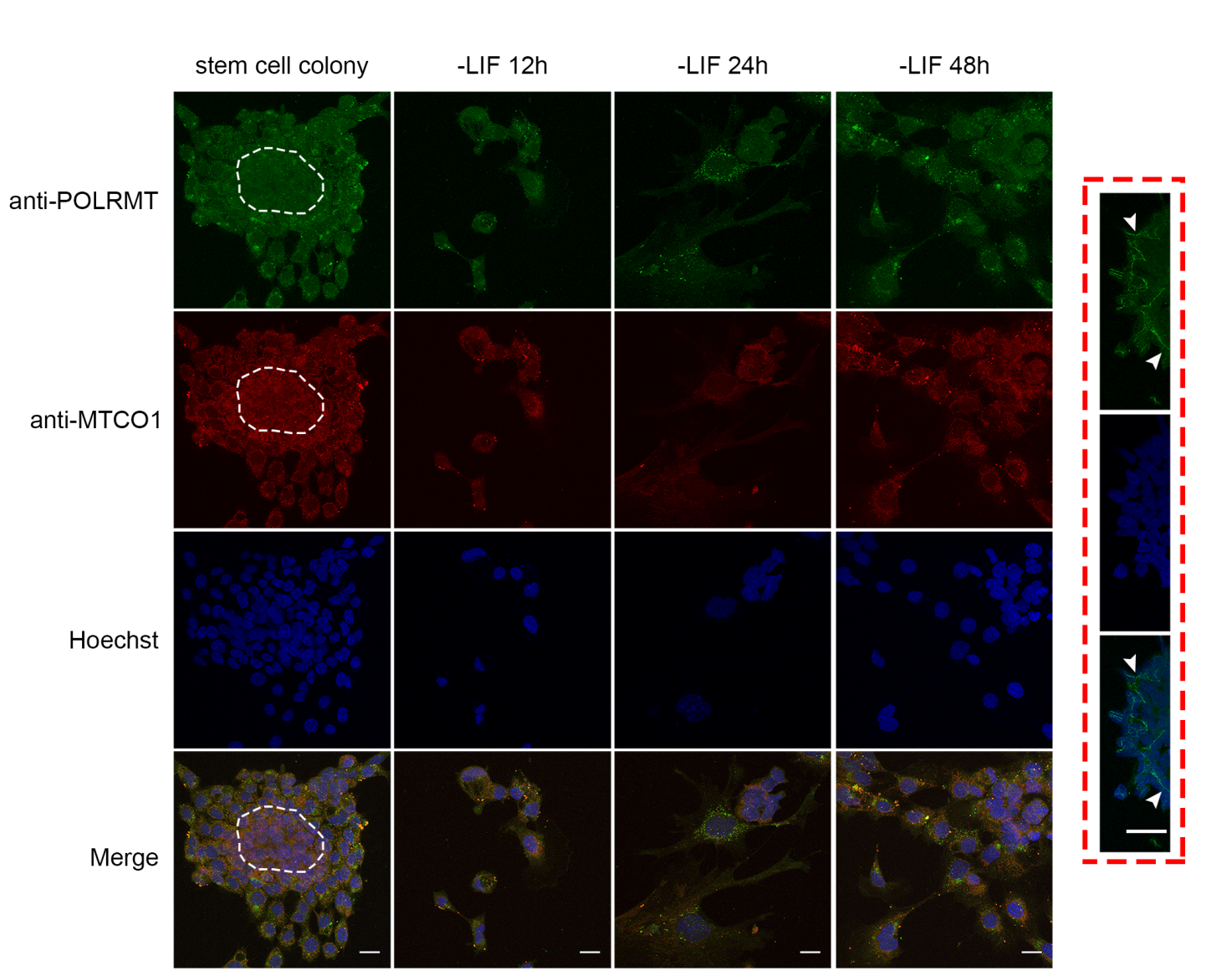


**Figure S10: POLRMT and MTCO1 expression increase during mammalian stem cell differentiation.** Staining of J1 mammalian cells with antibodies against POLRMT and MTCO1. Low expression of both proteins can be detected at the core of the stem cell colony (white circle) and higher in the periphery. Upon LIF removal, as the cells progress towards differentiation, an elevation of the expression of both proteins can be observed. In parallel, mitochondria with elongated shape (red rectangle, arrowheads) can be visualized. Images were acquired using a X40 objective lens. Scale bars, 20μm.
